# An assessment of nurses’ perceived and actual household emergency preparedness

**DOI:** 10.1371/journal.pone.0300536

**Published:** 2024-04-18

**Authors:** Gavin David Brown, Caroline McMullan, Ann Largey, David Leon

**Affiliations:** DCU Business School, Dublin City University, Dublin, Ireland; Sichuan University, CHINA

## Abstract

Nurses’ household preparedness is critical if they are to avoid role conflict and report for duty during an emergency. To date, the alignment between nurses’ perceived and actual household preparedness remains under examined. Investigating one of these variables in isolation fails to consider that perceived and actual household preparedness must be high and aligned. If misaligned, vulnerabilities could surface during emergencies, like concerns about family safety, potentially impacting a nurse’s commitment to duty during a crisis, or nurses may lack the actual preparedness to continue working long hours during an emergency. An online questionnaire was distributed to registered nurses in Ireland. The questionnaire was informed by a review of the literature and captured nurses’ perceived and actual household preparedness, attitudes towards and exposure to a range of emergencies, and pertinent demographic characteristics. The results showed a relationship between how nurses view their household preparedness and their actual preparedness. Regression analyses indicate that while there is an overlap, the factors associated with how prepared nurses think they are and how prepared they are can differ. This means that strategies to boost actual preparedness may differ from those needed to boost perceived preparedness. This finding underscores the importance of psychosocial preparedness. Feeling prepared is crucial as it can influence how one responds in an emergency. Considering both the perceived and actual aspects of household preparedness can lead to a more effective response during emergencies.

## 1. Introduction

At few times in history has the importance of the role played by nurses been so evident as during the COVID-19 pandemic. Having the correct number of nurses with the required skills mix is vital to a successful response to health-related emergencies or where healthcare is required because of other emergencies. When an emergency occurs, nurses must leave their homes to fulfil a critical role in the response. Nurses are, however, less likely to report for duty if they feel that doing so increases the risk to their own family or household. For this reason, there is some expectation that frontline personnel are personally as well as professionally prepared in advance of any emergency. FEMA [1] and Landahl and Cox [2] emphasized the importance of frontline personnel being prepared at home if they are to avoid role conflict: “Without taking the appropriate steps to prepare themselves and their families in advance of a disaster, responders will be hindered in their ability to perform their jobs when a disaster strikes, and will instead be focused on personal and family safety” [1]. Extending this further, Balut et al.’s [3] research found that healthcare workers who have family emergency plans are more likely to have confidence in their facility’s emergency response capabilities and are more likely to view their role as important in responding during an emergency, such as a pandemic. Research has shown, however, that frontline service personnel, including nurses, are not consistently prepared at home [4, 5] and that household preparedness is as important as workplace preparedness [6–10]: Corwin et al. concluded that “response professionals largely mirror laypeople in terms of their household preparedness levels” [4], while Uhm et al. [6] stress that to enhance response capacity of EMTs, and other frontline staff, education/training to promote self-efficacy and personal preparedness is required.

The emergence of role conflict among frontline personnel and its impact on an individual’s intention to report for duty during an emergency has been investigated extensively [11–13]. Much of this research focused on healthcare professionals and revealed that home and family concerns presented as key barriers to reporting for duty [14–37]. These barriers included concern for personal and/or family safety [14, 16–19, 23–27, 30, 34–39] transport worries, finding childcare, supporting elderly parents, and/or pet care [16, 17, 33, 35, 40, 41]. Slepski [42] reported that several respondents (nurses, physicians, emergency medical technicians) who responded to Hurricane Katrina and/or Hurricane Rita “mentioned the need to be personally ready … ensuring that their families were equally prepared … and that those left at home needed to be able to be ‘on their own’”.

Trainor and Barsky’s research [12] on role conflict, strain and abandonment among frontline personnel when faced with an emergency, recommended developing a family support and safety framework similar to the employee household preparedness program proposed by FEMA [1]. Any measure which drives preparedness is to be welcomed given that research has shown that actual preparedness resulted in individuals/homes being more resilient, which in turn increased nurses’ willingness to report for duty during an emergency [15, 43]. Connor [43] found that healthcare workers’ intention to report to work reflected personal preparedness measures such as “vaccinations, supplies, evacuation, and contact plans”. In essence, if their household had undertaken emergency preparedness planning activities, emergency medical services workers were found to be “more than twice as willing to mobilize to another, more severely affected community” [18].

While a number of studies have examined nurses’ emergency household preparedness, none have compared how prepared nurses feel (perceived preparedness) and their actual preparedness (checklist preparedness) [28, 30, 44–47]. Wilcox et al. [45] in their analysis of household emergency preparedness, highlight that nurses should prepare emergency overnight bags in case they are unable to leave the hospital due to severe weather or other emergencies. They should arrange, where necessary, backup carers for children or pets in the event of extended shifts or being unable to return home safely [45]. Balut et al. [44] found that USA healthcare workers who felt they were not prepared at a household level for major emergencies were more open to receiving preparedness training. They stressed that there was a need to “consider personal preparedness training that encourages [healthcare] employees to put together basic disaster kits at home as well as written household disaster plans that address the needs of dependents and others whom they may be responsible for in the event that they have to report to work during a disaster” [44]. Labrague et al. [48] also confirmed that workplace training has been shown to significantly enhance personal preparedness. Furthermore, the training of nurses in emergency preparedness can have a knock-on effect on the preparedness of those under their care. This impact was illustrated by in a paper focused on community health nurses [49].

While several studies have examined nurses’ emergency household preparedness [3, 28, 30, 42, 44–52], comparing how prepared nurses feel (perceived preparedness) and their actual preparedness remains underexamined, with Longo’s [52] study of student nurses a rare exception. Even when widening the scope to include the general population, only a few studies have examined the alignment of perceived and actual household preparedness [52–59]. For example, Kapucu [57] established that while 61% of respondents felt adequately prepared for an emergency, only 8% had emergency supply kits with three days of food, water and medications. Similarly, Ablah [59] observed that 66.5% of respondents who rated their perceived household preparedness as “somewhat well prepared” or “well prepared” were found to be “unprepared” meaning they were missing 2 or more of the 6 measures of preparedness: an evacuation plan; 3-days of water; 3-days of food; 3-days of medication; radio; flashlight.

This paper examines perceived and actual emergency preparedness of nurses at a household level. Perceived preparedness is a subjective assessment of household preparedness based on how prepared the respondents feel [53]. When measuring perceived preparedness, some studies use a binary measure [55, 60, 61]. For example, Cope et al. [61] asked respondents if they thought they were prepared to deal successfully with a serious threat: allowing respondents to indicate yes or no. Other studies use ordinal scales [54, 56, 62–69] that vary from three-point scales [54, 56, 64], to four-five points scales [63, 65, 69], and larger [62, 66]. For example, DeBastiani et al. [64] asked respondents how well prepared they felt their household was to handle a large-scale emergency: allowing respondents to indicate “well prepared, somewhat prepared, not prepared at all”.

Determining actual preparedness involves asking the respondent if one or more measures to prepare for an emergency have been undertaken within the household [53]. These measures are defined by taking account of the needs of the household when faced with an emergency [57] and are commonly drawn from preparedness guidelines [4, 55, 59, 70–74] or previous preparedness studies [66, 75–79]. When measuring emergency preparedness, some studies use a single binary measure [47, 70], for example, asking respondents if there is a 72-hour emergency kit in their home [47]. To maintain measurement validity, household preparedness measures should include measures which are not focused solely on supplies [58], because as Mileti [80] noted “the purpose of preparedness is to anticipate problems in disasters so that ways can be devised to address the problems effectively and so that the resources needed for an effective response are in place beforehand”. Preparedness measures can be grouped into categories such as:

- emergency resources, equipment and supplies such as a first aid kit and fire extinguisher [81], a disaster survival kit [70], or radio with spare batteries [76].
- planning measures [4, 55], such as creating a communication plan [47] or gathering important documents [76], and
- preparedness actions [72, 82] such as buying insurance [83] or securing furniture [54, 77].

In some cases, these preparedness measures are then compiled into scales [60, 74, 76, 77, 79, 82–90], with each measure equally weighted and summed [54, 83–85, 90–92]. For example, DeYoung and Peters [79] used an adapted version of the Mulilis-Lippa Earthquake Readiness Scale, which contained 12 equally weighted measures: meeting place, fire extinguisher, decide where to live, water, battery radio, food, medicine, cooking source, CPR training, first aid, attended preparedness meeting, and flashlight. Others have chosen to examine each measure separately [55, 56, 78, 92, 93]; for example, Hung [78] studied specific preparedness behaviors that included having an electric generator, evacuation plan, emergency contact and insurance.

The motivation for this study was twofold: firstly, while studies associated with frontline responders’ household preparedness have been undertaken [2, 4, 5], there has been a call for more research on nurses’ household preparedness [94, 95]. For example, Baduge et al. [94] stressed “the personal preparedness of emergency nurses has been under-examined and requires more evidence for strategy”. In Ireland, the setting of this study, McMullan et al. [96] found that nurses’ homes were underprepared for influenza outbreaks; however, limited data was available on household preparedness for other types of emergencies. Secondly, a review of the literature examining both perceived and actual household preparedness, revealed no comparison of nurses’ perceived and actual household preparedness had been undertaken. See the S1 Table for a summary of the literature review and its outputs. Household preparedness is an important foundation for resilience. If there is a mismatch nurses’ perceived and actual household preparedness this may have significant negative effects on nurses’ capacity to work during an emergency. For instance, during Storm Emma, which hit Ireland from 28 February to 4 March 2018, there were extensive disruptions that greatly affected transport, water, power, and food supplies. During the emergency, some nurses could not get to work due to the weather or did not want to leave their homes because of competing priorities. Others, who reported for duty, were unable to return to their homes. These nurses faced difficult conditions, often sleeping in crowded spaces with limited facilities, privacy and access to hot food [97]. Storm Emma emphasized the need for greater personal emergency preparedness supports and for nurses.

The outputs from the S1 Table also demonstrated that risk rating, prior exposure to emergencies, and a set of socio-demographic factors were commonly used in studies of perceived or actual preparedness, but the extent to which these factors align in the influence of perceived and actual preparedness remained unclear. For example, Basolo et al. [55] found that owning a home had a significant positive effect on stocking emergency supplies but had no significant effect on levels of perceived preparedness. To address these gaps in the research, the following research questions were examined:

1. How aligned are nurses’ perceived and actual household preparedness?
2. To what extent do risk rating, prior exposure to emergencies, and socio-demographic factors influence perceived and actual preparedness among nurses?

By targeting this specific group, our study brings a unique perspective to the field and contributes to a better understanding of the household preparedness of nurses’ who are pivotal in emergency response. This assessment of perceived and actual household preparedness provides a more nuanced understanding of the factors influencing nurses’ preparedness. This knowledge can guide tailored interventions to enhance their preparedness for future emergencies.

We expected differences between perceived and actual preparedness to map the research into risk perception which has shown subjective perceptions of risk do not match objective/expert risk assessments [73, 90–97]. For example, Slovic et al. [98] showed that the public’s perception of risk was often based on feelings, suggesting “risk as feelings refers to our fast, instinctive, and intuitive reactions to danger”; as opposed to the mode of thinking known as “risk as analysis” which “brings logic, reason, and scientific deliberation to bear on hazard management” [98]. Following this logic, nurses’ perceptions of their household preparedness will be based on preparedness as a feeling. This is different to actual preparedness, where the person completes an assessment against a given checklist of items and actions; thereby reducing subjectivity.

**Fig 1:**
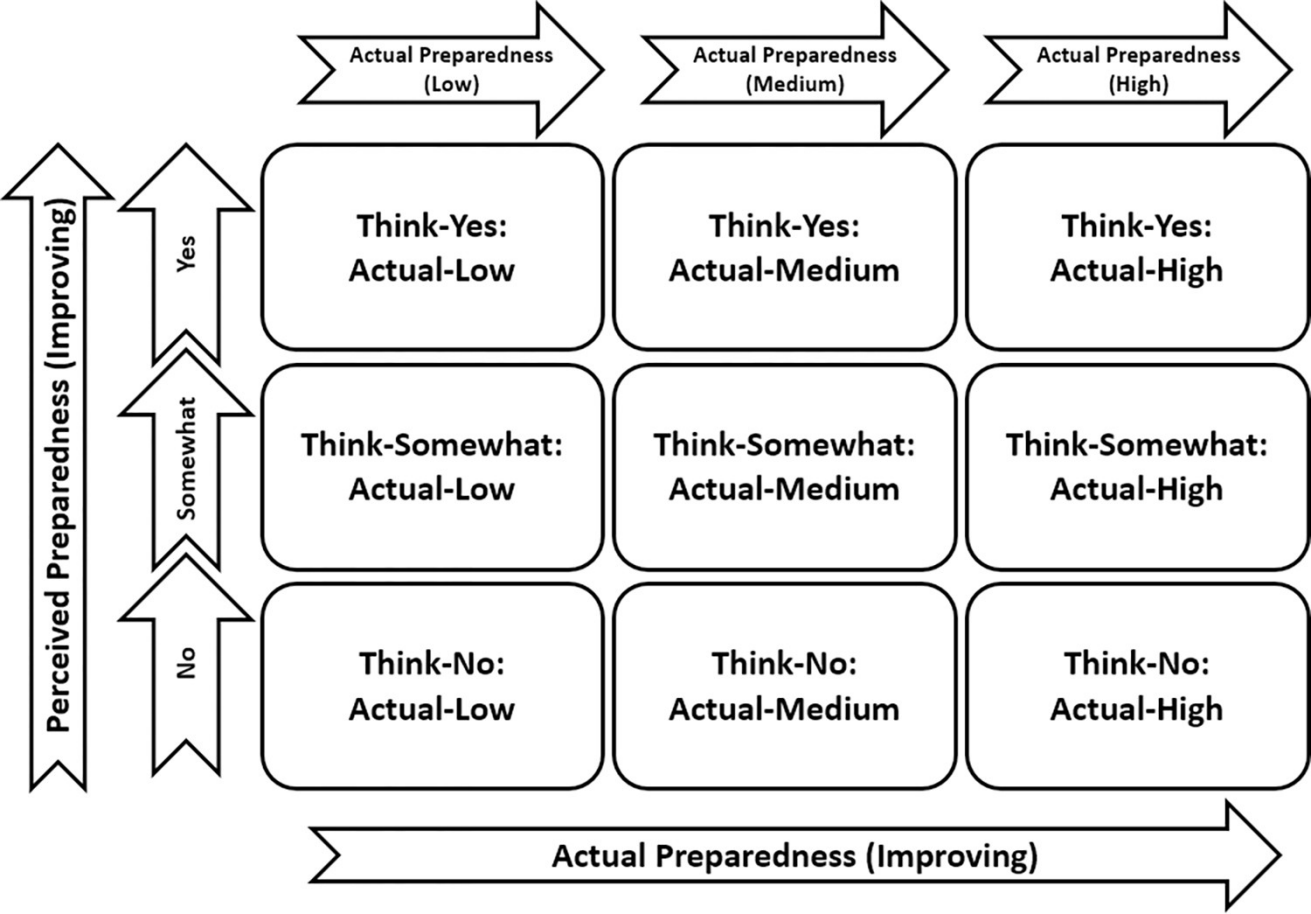
The possible combinations of perceived and actual preparedness.

Risk compensation theory suggests those who perceive a reduced level of risk are less likely to adapt their behavior and become less careful because they feel more protected [98–100]. Similarly with preparedness, a high level of perceived preparedness may result in less protective action being taken. Unless this high level of perceived preparedness is matched by a high level of actual household preparedness, vulnerabilities may emerge during an emergency. Given that home and family concerns can present as key barriers to nurses reporting for duty, the effects of not having perceived and actual household preparedness high and aligned could be detrimental to the continuity of services during an emergency. Informed by Kyne et al. [53], Fig 1 illustrates the possible combinations for perceived and actual preparedness, with the optimum outcome being Think-Yes: Actual-High.

Role conflict is most likely to occur when a nurse falls under one of the “Think-No” categories. In these instances, a nurse perceives they have low household preparedness and that leaving home during an emergency may put their household at additional risk; as a result, they may choose not to report for duty. Role conflict may also occur when a nurse falls under one of the other “Actual-Low” categories. For example, if a nurse leaves their home thinking their household can cope with the impending emergency, but later finds they were unable to cope due to a lack of actual preparedness, they may decide not to return to work the following day.

A decision not to report for duty is especially critical in Ireland where there are already low nursing staff numbers. For example a Health Information and Quality Authority’s (HIQA) report on University Hospital Limerick in March 2022 found “nurse staffing levels were insufficient, which was having an impact on the safe provision of care at the time of inspection” [101].

## 2. Literature review on perceived and actual preparedness

Ten studies examined the empirical differences between perceived preparedness and actual preparedness [52–56, 58, 59, 64, 77, 102]. Longo, who utilized dichotomous variables for perceived and actual preparedness, and odds ratios to undertake the analysis, discovered that nursing students who felt prepared for earthquakes were in fact more than twice as likely to have a family emergency plan in their household, and nearly five times as likely to have accumulated emergency supplies [52]. Studies which examined these differences among the general population using correlation analysis reported relatively weak positive correlations between perceived and actual preparedness [54–56], which ranged from r = 0.156 to r = 0.401. Basolo et al. [55, 56] reported that perceived preparedness was not correlated with some measures of actual preparedness, such as having emergency supplies [55]. In a 2020 study, Kyne et al. [53] defined respondents as being prepared for an emergency if they had eight or more items from a checklist of nine and perceived themselves as prepared if they stated they were at least somewhat prepared. Based on a paired t-test analysis, significant differences between perceived preparedness and actual preparedness were reported. The remaining studies examined actual preparedness using regression analysis, with perceived preparedness as an explanatory variable and controlling for other factors. The results were mixed [59, 64, 77, 102]. Nguyen et al. [77] found the association between perceived and actual preparedness non-significant. They suggested, however, that respondents who “had done some of the ‘easier’ things” relating to earthquake preparedness, such as gathering preparedness items like canned food, bottled water, and a first-aid kit, and who perceived they were prepared were less likely to undertake any further preparedness actions [77]. In contrast, Rebmann et al. [102], DeBastiani et al. [64], and Ablah et al. [59] found that perceived preparedness was positively and significantly associated with actual preparedness. For example, DeBastiani et al. [64] suggested that people who reported feeling well prepared were 4.2 times more likely to have a 3-day supply of food kept in the home, but this association reduced to 2.3 times more likely to have a flashlight. Table 1 provides a summary of the perceived and actual preparedness measurements used along with the findings related to their association. The combined findings from these studies suggested that assessments of perceived preparedness to some extent reflected actual household preparedness, but the association is neither consistent nor strong.

**Table 1:** Association between perceived and actual preparedness.

Source	Analysis	Emergency	Measurement of Actual Preparedness	Measurement of Perceived Preparedness	Result
Longo [52]	Odds ratios	Earthquake	Has a family plan or not (dichotomous)	Felt prepared vs Felt unprepared	2.75 times more likely to have a plan**
			Has emergency supplies or does not (dichotomous)		4.83 times more likely to have supplies***
Basolo et al. [55]	Correlations	Earthquake	Has a family plan or not (dichotomous)	Perceived level of preparedness 1 = high level; 0 = low level	r = .33*
			Has all 11 items (supplies) or does not (dichotomous)		NS
			Did three mitigation measures or not (dichotomous)		r = .21*
			Knows how to shut off utilities or not (dichotomous)		r = .25*
		Hurricane	Has a family plan or not (dichotomous)	Perceived level of preparedness 1 = high level; 0 = low level	r = .23*
			Has all 11 items (supplies) or not (dichotomous)		r = .18*
			Knows how to shut off utilities or not (dichotomous)		NS
Basolo et al. [56]	Correlations	Hurricane	Intend to evacuate or not (dichotomous)	Perceived household preparedness 3-point scale: low-to-moderate, moderate-to-high, and fully prepared.	NS
			Availability of sandbags (dichotomous)		NS
			Has a family plan or not (dichotomous)		r = .156**
			Has all 11 items (supplies) or does not (dichotomous)		r = .279**
DeBastiani et al. [64]	Adjusted Odds Ratio	Public Health Emergencies	Has 3-day supply of food	Perceived household preparedness: well prepared, somewhat prepared, not prepared at all	Respondents who reported feeling their households were well prepared were: 4.2 times more likely to be prepared
			Has 3- day supply of water		4.0 times more likely to be prepared
			Has written evacuation plan		3.1 times more likely to be prepared
			Has working battery-operated radio and batteries		2.6 times more likely to be prepared
			Has working flashlight and working batteries		2.3 times more likely to be prepared
			Number of preparedness items—increase		As the number of preparedness items increased, respondents were more likely to report feeling their households were well prepared: 0 items, 4.2%; 1 item, 4.2%; 2 items, 8.2%; 3 items, 15.4%; 4 items, 35.1%; 5 items, 56.5%
Kirschenbaum et al. [54]	Correlations	Earthquake	Actual preparedness: 17-items scale	1 = not prepared at all; 3 = fully prepared	r = .401**
Kyne et al. [53]	Paired t-test	Hurricane	Actual preparedness: nine action statements	1 = not prepared at all, 2 = somewhat well prepared, 3 = well prepared	t = 2.9814**
Nguyen et al. [77]	Logistic Regression	Earthquake	preparedness activities—some (1–5) vs. no preparation	Perceived pre-quake preparedness: 0 = unprepared; 3 = well prepared	NS
			preparedness activities—many (6 or more) vs no preparation		NS
Rebmann et al. [102]	Linear Regression	"Natural Disasters" and Pandemics	Disaster Preparedness (20 indicators)	Perception of personal preparedness for:	"Natural disaster" β = 3.1 ***, S.E. (0.48) "Pandemic" β = 1.8 ***, S.E. (0.51)
			Pandemic Preparedness (9 Indicators)		"Natural disaster" β = 3.0 ***, S.E. (0.24) "Pandemic"β = .7 **, S.E. (0.51)

Notes

*p < .05

**p < .01

***p < .001, NS = not significant

Differences in the level of emergency preparedness can be attributed to “human, social, economic and demographic factors [that] influence the ability and desire of individuals and families to prepare for emergencies” [103]. Similarly, social and demographic factors, along with experiences of risk, have been found to play a major role in shaping risk perception [104]. The wide range of factors that have been examined in relation to perceived and actual preparedness, and the methods employed, are reported in the S2 Table. While the number of papers is relatively small, the findings highlight differences in the factors that predict both perceived and actual preparedness.

Table 2, which summarizes the factors that were included in the analyses of perceived and actual preparedness, shows inconsistencies in the selection of demographic factors between studies, with Basolo et al. [55, 56] using the most comprehensive range of socio-demographic factors. Basolo et al. [56] found that age was significant and positive for perceived preparedness. The presence of children in the home, home ownership, identifying as male, and marital status were significant and positive for at least one actual preparedness variable (having a family plan, having sandbags on-site, or having all recommended preparedness items on hand). Kirschenbaum et al. [54] showed that gender (being female) and age were significant and positive for perceived preparedness, while education was non-significant. For actual preparedness, this was reversed, with gender and age becoming non-significant and education becoming significant and positive.

**Table 2:** Variables included in both the perceived and actual preparedness regressions.

	Basolo et al. 2009 [55]	DeBastiani et al. 2015 [64]	Henly-Shepard, et al. 2015 [60]	Basolo et al. 2017 [56]	Kirschenbaum et al. 2017 [54]	Ranjbar et al. 2018 [66]
**Emergency (Context)**	Hurricane & Earthquake	Public Health Emergencies	Natural Hazards	Hurricane	Earthquake	Earthquake
**Gender**	Male/ Female	Male/ Female	Male/ Female	Male/ Female	Male/ Female	
**Age**	Age in years	Age in categories		Age in years	Age in categories	
**Children**	Child present in home			Child present in home		
**Income**	Ranges from less than $10,000) to greater than $120,000 (Interval)		Categories: Low, Medium and High	Ranges from less than $10,000) to greater than $120,000 (Interval)		
**Race**	White/Other	White non-Hispanic Black non-Hispanic Hispanic Other non-Hispanic		White/Other		
**Home**	Home Ownership Tenure/Other			Home Ownership Tenure/Other		
**Household size**						Number of family members
**Years of residence**			< 1 year residence 1–5 years residence 5–10 years residence 10–20 years residence			
**Marital status**	Married/Other			Married/Other		
**Region**			Origin: Mainland Origin: Kauai Origin: Hanalei			Residence region
**Religion**					Jewish	
**Education**	Graduated from high school	Less than high school; High school diploma; Some college; College or more.		Graduated from high school	Educational status: academic	
**Immigrant**				Not born in the United States		
**Hazard Exposure (experience)**	Experienced major earthquake Experienced major hurricane			Experienced major hurricane	Experience	Experience
**Risk**	Dread Fatal Happen			Risk Perception	Living in risk zone	
**Confidence in relevant officials**	High level; Low level			No confidence to total confidence (scale)		Trust in Emergency authorities
**Access to relevant guidance**	Number of Disaster Preparedness Information Sources used (scale)		Know of public awareness programs Think early warning systems are effective	Received some emergency preparedness information Received a lot of emergency preparedness information	Trust in information source	
**Community**			Participation in a Community Group Feel the community is prepared			

Note: Factors that were not tested in both regressions are not listed.

The literature review by Wachinger et al. [105] suggested experiencing an emergency had an effect on promoting household preparedness. An examination of the models in S3 Table revealed that the significance of exposure to emergencies was inconsistent depending on the preparedness measure. For example, Basolo et al. [56] found that exposure to hurricanes significantly and positively impacted family emergency planning, but no other actual preparedness measure (for example, having sandbags on-site) or perceived preparedness. Kirschenbaum et al. [54] similarly found that exposure to earthquakes positively and significantly affected actual household preparedness but not perceived preparedness.

Wachinger et al. [105] noted that while people may report high risk perception scores, this did not necessarily translate into preparedness action. Bourque [106] suggested that “although risk perception may be a necessary predictor of preparedness, it is not a sufficient predictor and is, in fact, largely mediated or moderated by other factors”. As can be seen from Table 2, only two studies, both by Basolo et al. [55, 56], included a measure for risk. Their analyses, however, did not identify a discernable pattern in the significance of risk perception on perceived and actual preparedness (see S3 Table for their results). For example, Basolo et al. [56] found that higher levels of risk perception positively and significantly influenced perceived preparedness and having sandbags on-site (actual household preparedness) but were non-significant for other actual household preparedness measures.

The remaining factors within Table 2 were considered more relevant to a single risk, or one classification of risk, rather than all-hazard studies (confidence in relevant officials, access to relevant guidance, and community) or in a more multi-cultural society than Ireland (race, religion, immigrant).

## 3. Methodology

### 3.1 Questionnaire design and dissemination

This study examined the household emergency preparedness of State Registered Nurses in Ireland. The questionnaire asked nurses to rate a range of risks drawn from the Irish National Risk Assessment (NRA), measured their perceived and actual household preparedness, identified exposure to a range of emergencies, and captured a variety of demographic characteristics.

To test the reliability, content validity and face validity of our measures, we pilot tested with a smaller, similar group of specialists working in emergency management and registered nurses. This allowed us to gauge the clarity (where they correctly understood), appropriateness, and relevance of the questions [107].

Under our university regulations, this study qualified as a low-risk social research project and was approved by the Research Ethics Committee at DCU Business School. Participants were over the age of 18, their participation was voluntary, and the data were collected using an anonymous online questionnaire. Before commencing the questionnaire, participants were provided with a plain language statement explaining the purpose of the study and offered an opportunity to contact the research team with any questions. To consent, participants clicked a button on the online questionnaire indicating they read the plain language statement and consented to take part in the study. It was made clear to participants that once their responses were submitted, they could not withdraw from the study, given the anonymous nature of the online questionnaire.

According to the Central Statistics Office (CSO) Ireland, there were 41,077 registered nurses working in the country at the time of data collection [108], 35,873 of whom worked for the Health Service Executive (HSE) in 2016 [109]. A clustering approach was taken to draw the sample, where a link to the questionnaire was circulated online in May and June 2016, across the Health Service Executive (HSE) email system. It was also posted on the Nurses and Midwives in Ireland private Facebook group which had approximately 15,000 members at that time to capture those not working in the HSE. Using the Qualtrics sample size calculator [110] to achieve a confidence level of 95% with a 5% margin of error required ideal sample size was 381, and we achieved a sample of 557 nurses. We also ensured proportionate representation of subjects based on gender; the CSO Ireland noted that in 2016 8.2% of registered nurses were male [108]. Our respondents are consistent with this gender breakdown, with 9.1% of respondents being male.

### 3.2 Variables: Perceived and actual preparedness

Perceived preparedness was captured early in the questionnaire by asking respondents, in the context of household preparedness, were they prepared to deal with an emergency using a 3-point scale (No, Somewhat, or Yes) aligning with DeBastiani et al. [64]. Actual preparedness measured the number of emergency resources, planning measures and preparedness actions a respondent reported. As our study was based in Ireland, we used 24 items from the Irish preparedness guidelines [111] and checked these against other established guidelines prepared by FEMA and the American Red Cross to ensure commonality [1, 112–114]. Use of the Irish list of items was in line with the Kohn et al [115] recommendation that studies of household preparedness should use the relevant national guidelines to assess preparedness.

A Household Preparedness Score was calculated by merging the three constructs (resources, planning, and actions), which was comparable with the American Red Cross preparedness campaign “Get a Kit; Make a Plan; Be Informed” [113] and the work of Corwin et al. [4]. This resulted in 24 indicators of preparedness (see Table 4). To achieve equal standardized weighting, the scores for resources, planning and action were z-transformed separately [4], which allowed the scores to be compared to one another more accurately as the number of items within each construct would not inflate the score. These standardized z-scores were averaged to give the Household Preparedness Score. This resulted in the following minimum and maximum scores for each scale:

- Preparedness Planning (a 5-item construct) min = -1.92; max = 2.13
- Preparedness Action (a 3-item construct) min = -1.90; max = 1.17
- Preparedness Resources (a 16-item construct) min = -2.63; max = 2.23
- Household Preparedness Score (the combined 24-item construct) min = -1.82; max = 1.84

The four preparedness indicators were checked for reliability and validity using Cronbach’s α and Spearman correlation coefficients with acceptable results. Kohn et al. [115], in their review of emergency preparedness studies reported a Cronbach’s α variance of between α = .42 and α = .99 depending on the study and items selected. The results of our tests are Preparedness Planning α = .55; Preparedness Action α = .47; Preparedness Resources α = .73; Household Preparedness Score α = .80. The correlations showed that all four preparedness indicators were significantly (p < .01) and positively correlated with one another. Individual correlations ranged from weak for “action–planning” (r = .362, p < .01), to strong for “Household Preparedness Score–planning” (r = .820, p < .01).

### 3.3 Variables: Risk rating, exposure, self-efficacy and socio-demographic variables

Respondents’ prior exposure to, and risk rating of, 17 risks from the NRA were gathered [116, 117]. Aligned with the NRA, the 17 risks were clustered into three risk groups:

- civil: foodborne disease outbreak, infectious disease affecting humans, infectious disease affecting livestock, loss of critical infrastructure, terrorism, and waterborne disease;
- socio-natural: drought, flooding, high temperatures, low temperatures, snow, and storms;
- technological: cyber incidents, disruption to the energy supply, fire, nuclear incident (abroad), and radiation (domestic).

Nurses’ exposure to prior emergencies was measured by asking whether they had experienced each of the 17 risks as emergencies using a self-reported yes/no measure. Responses were summed by classification to create three measures each ranging from 0 to 6: exposure to socio-natural emergencies, which had a mean 2.49 with a standard deviation of 1.47, technological emergencies, which had a mean 1.07 with a standard deviation of 0.76, and civil emergencies, which had a mean 1.34 with a standard deviation of 1.38.

Risk rating was measured using the five-point likelihood and impact Likert scales from the Irish NRA [116], which was identified, from the literature review (S1 Table), as a common method used to measure risk [69, 71, 83, 91, 92, 118]. These scales are multiplied to give a risk rating score for each risk which ranged from 1 to 25. To provide a measure of risk rating for each risk category either an average value across the risk category or the maximum risk rating value from within the risk category can be used. The average risk rating measure for each risk classification is calculated as the arithmetic mean of risk ratings for risks within the classification. The model was run twice, once using the average risk ratings for each classification and a second time using the highest risk ratings. This allowed us to examine whether the highest risk score within the risk classification influences perceived and actual preparedness (using maximum risk) or a combined risk rating for the risk classification (using mean risk). The outputs of the regression models were compared, and no changes in significance, the direction of the effect, or meaningful change in the size of the effect were found. As a result, we report our findings using the outputs from the average risk rating for each classification and provide the models using the highest risk ratings in the S3 Table. This results in three averaged risk rating variables for each model: socio-natural risks, which had a mean 8.38 with a standard deviation of 3.21 and a Cronbach’s α value of .80; technological risks, which had a mean 11.51 with a standard deviation of 3.79 and a Cronbach’s α value of .78; civil risks which had a mean 11.62 with a standard deviation of 4.41 and a Cronbach’s α value of .89.

Self-efficacy gauges an individual’s perceived capability to cope with an emergency. It is assessed by inquiring if respondents believed they would need help from neighbors, non-profit organizations, emergency services, local, or national governments during an emergency. Respondents rated each of the six assistance sources on a scale where "no assistance required" scores a 6 and "substantial assistance required" scores a 1. A self-efficacy measure is derived by averaging the five scores, with 1 being the lowest score and 6 the highest. The mean score for self-efficacy stands at 3.40, with a standard deviation of 1.19 and a Cronbach’s α value of .87.

The literature reviews by Levac et al. [103] and Kohn et al. [115] concluded that the effects of socio-demographic factors differ across studies. However, as noted in the literature review, it was unclear if the preparedness measure used may have contributed to this variance. At the time of the study, Basolo et al. [55] analysis of perceived and actual preparedness was most extensive and had examined gender, marital status, annual household income, age, race, children, education, owns the home. As the respondents were nurses, all of whom had a tertiary qualification, the education factor was excluded and instead working full-time, and years of experience were included. Cohabitation was captured using the number of adults living in the household. Finally, the data from a previous Irish study showed that urbanicity was significantly related to actual household preparedness [58]; hence this variable was included. A detailed summary of respondent demographics is presented in Table 3.

**Table 3:** Summary characteristics.

Characteristic		Description
Gender (430n)		
Female	90.9% (391n)	Coded 1 if female, 0 otherwise
Aged (430n)		
34 or under	32.8% (141n)	Coded 1 if aged 34 or under, 0 otherwise
35–44	40.2% (173n)	Coded 1 if aged 35–44, 0 otherwise
45 or older	27% (116n)	Coded 1 if aged 45 or older, 0 otherwise
Lives in a: (430n)		
City	14.2% (61n)	Coded 1 for city, 0 otherwise
Suburb/city outskirt	17.2% (74n)	Coded 1 for Suburbs, 0 otherwise
Town	26.7% (115n)	Coded 1 for town, 0 otherwise
Village	13.5% (58n)	Coded 1 for village, 0 otherwise
Rural area	28.4% (122n)	Coded 1 for rural area, 0 otherwise
Own vs Rent Home (430n)		
Own Home	74.2% (319n)	Coded 1 for homeownership, 0 otherwise
Years living at current address (427n)		
Mean (SD)	9.94 (8.46)	Variable Ranged from 1 to 57
Adults (age>18) living at the address (428n)		
Mode	2	Variable Ranged from 1 to 7
Children (age<18) living at the address (419n)		
Mode	0	Variable Ranged from 0 to 5
Household Income (360n)		
Below 30,000	8.6% (31n)	Coded 1 for income below 30,000, 0 otherwise
30,000–70,000	64.2% (231n)	Coded 1 for income 30,000–70,000, 0 otherwise
Over 70,000	27.2% (98n)	Coded 1 for income over 70,000, 0 otherwise
Nursing Work (430n)		
Full-time nurse	79.3% (341n)	Coded 1 if working full-time, 0 otherwise
Years of experience (393n)		
Mean (SD)	14.59 (9.17)	Variable Ranged from 1 to 42

As outlined in the literature review, and evident in the S1 Table, the significance and direction of influence of perceived and actual preparedness are both varied and inconsistent across studies.

### 3.4 Quantitative analysis: Descriptive and inferential statistics

The analysis was carried out using the statistical software package STATA (StataCorp; Release 14.2/SE). To conduct essential model diagnostics, we initially inspected the correlation matrices to identify possible multicollinearity and to examine the pairwise relationships between the variables. Subsequently, we employed the variance inflation factor (VIF) to determine the extent to which the variance of a given regression coefficient was inflated by collinearity.

In line with the research questions, the analysis was divided into stages. First, descriptive statistics were used to examine nurses’ perceived and actual household preparedness. Chi-square tests of association, Spearman’s correlations, and a partial generalization of ordered probit analysis with marginal effect calculations were then used to examine how the measures for nurses’ perceived and actual emergency preparedness were related. In line with research question one, the hypothesis was to test whether a positive association existed between perceived and actual preparedness.

Hypothesis 1: There is a significant positive alignment between nurses’ perceived household preparedness and their actual household preparedness.

The second stage of the analysis addressed research question two. Regression techniques were employed to examine the relationship between risk rating, prior risk exposure, socio-demographic factors and the dependent variables of perceived and actual preparedness. These regressions are summarized in the following function:


Preparedness(Y)=f[riskrating(socio–natural,technological,civil),exposuretoemergencies(socio–natural,technological,civil),yearspracticing,socio–demographics]

Based on the literature, it was hypothesized:

1. Hypothesis 2: Risk rating is positively and significantly associated with higher levels of perceived and actual preparedness. High risk ratings suggest awareness of risks and potential vulnerability, which should prompt preparedness action.
2. However, there is a potential for reverse causality. Hypothesis 3: If a respondent has undertaken the identified preparedness measures, this may result in a lower assessment of risk [106]. If this is the case, we may see low levels of risk rating associated with high levels of perceived preparedness. This could lead to nurses not reviewing or revisiting their household preparedness or they may ignore public awareness campaigns designed to drive household resilience.
3. Hypothesis 4: Exposure to an emergency is associated with higher levels of actual preparedness as experiencing an emergency leads to the taking of measures to increase household preparedness.
4. Conversely, Hypothesis 5: direct exposure to emergencies may lead to a non-protective response (denial, fatalism) and feelings that it is not possible to protect against future emergencies. In such circumstances exposure to an emergency is associated with lower levels of perceived preparedness.
5. Hypothesis 6: Socio-demographic factors significantly impact both perceived and actual preparedness among nurses.

Actual preparedness was analyzed using a standard OLS regression. The results showed that the VIF scores were within acceptable limits (VIF <5) [119], having a maximum VIF of 3.71 and a mean VIF of 1.91. Furthermore, the analysis used robust standard errors to control for possible heteroscedasticity.

The ordinal measure of perceived preparedness has three categories, No, Somewhat and Yes, with the observed variable Y coded as 0, 1 and 2, respectively. Underlying the observed response, there is assumed to be a latent variable Y*, modelled as Y* = *Xβ*+*ε*, which is a continuous measure of preparedness perception. Two cut-off points μ_1_ and μ_2_ exist on the latent scale, with μ_1_ < μ_2_, such that Y = 0 if Y* ≤ μ_1_, Y = 1 if μ_1_ < Y* ≤ μ_2_ and Y = 2 if Y*> μ_2._ Thus, for example, it is assumed that an individual stated No, they are not prepared if their assessment of preparedness falls below the threshold μ_1._ At the other extreme, they state Yes, they are prepared when their underlying perception of their preparedness is high enough on the scale (Y*> μ_2_). This study examined perceived preparedness using a partial generalization of ordered probit that allows the impact of an independent variable to change over the underlying scale. The probability of each outcome is shown in the equation set (Eq 1) below:

$$\begin{array}{c} P(Y=0)=\Phi (\mu_{1}-X\beta_{1})\\ \;P(Y=1)=\Phi (\mu_{2}–X\beta_{2})-\Phi (\mu_{1}-X\beta_{1})\\ \;P(Y=2)=1-\Phi (\mu_{2}–X\beta_{2}) \end{array}\}$$ (Eq 1)

where *Φ* (.) is the cumulative normal distribution. This formulation differs from the standard ordered probit model, which invokes the parallel regressions assumption, where the coefficient matrices β_1_ and β_2_ are constrained to be equal. The user-written gologit2 command in STATA [120] with a probit link function was used, with the auto-fit option invoked to identify independent variables which violated the parallel regressions assumption of the standard ordered probit. The coefficients on these variables differ between matrices β_1_ and β_2,_ and in these cases, both values are presented in the results. Robust standard errors are computed in the analysis to address any concern of heteroscedasticity. Marginal effects, calculated at the means of independent variables, are also reported to indicate the magnitude of the effects.

## 4. Results

### 4.1 Nurses’ household preparedness

In the context of household preparedness, many nurses (60.8%) considered themselves to be somewhat prepared, or prepared (15.5%), while 15.3% stated no, they were not. The remaining 8.4% of nurses were unsure and were excluded from the remainder of the analyses.

Considering actual preparedness on the 24-item Household Preparedness Score, nurses scored between 3 and 24 items, with a mean value of 13.46 and a standard deviation of 4.36. The breakdown by specific item/task is given in Table 4, Preparedness Items and Actions. Over half of nurses had an emergency plan in place for a specific situation (57.1%), while 13.1% had an emergency plan that would cover all situations. Regarding resources in their home, less than half of the nurses had enough water for 3+ days (40.3%), and 45.6% had medications for 8+ days. On the other hand, more than three-quarters of respondents (85.9%) had enough food for 3+ days. In addition, 19.4% of nurses said they kept the resources solely for an emergency, and 39.7% said they regularly check these resources.

**Table 4:** Preparedness items and actions.

	% (n)	Total
**Planning**		
Emergency plan—all situations	13.1% (61)	467
Emergency plan—specific situations*	57.1% (267)	468
List of emergency contact numbers	47.9% (224)	468
Important documents kept in the home	84.6% (396)	468
Cash kept in the home	41.9% (196)	468
**Action**		
Acted to protect self or home in case of an emergency	60.2% (327)	543
Household insurance	82.5% (378)	458
Flood insurance**	51.7% (233)	451
**Resources**		
Smoke detector	99.4% (465)	468
Fire extinguisher	59.8% (280)	468
Fire blanket	55.3% (259)	468
Carbon monoxide detector	57.9% (271)	468
Generator	8.5% (40)	468
Gas Grill (with spare gas)	32.5% (152)	468
Tank of fuel (small can, etc.)	29.9% (140)	468
Tool set	82.3% (385)	468
Axe/Chain saw	53% (248)	468
First aid kit	89.3% (418)	468
Batteries	82.5% (386)	468
Torch	89.1% (417)	468
Battery powered radio	27.4% (128)	468
Enough water for 3+ days	40.3% (188)	467
Enough food for 3+ days	85.9% (402)	468
Enough meds for 8+ days	45.6% (213)	467

Notes

* In line with the Irish preparedness guidelines [94] a distinction is made between a plan for all situations and specific situations (e.g., house fire). Fifty-five nurses indicated they had a plan that covered specific situations in addition to an all-situation plan, while six indicated they had an all-situation plan, but did not have a plan for any specific situation.

** Flood insurance identifies those whose household insurance covers flooding.

Overall, 327 (60.2%) nurses reported taking at least one household preparedness action. Of the 216 nurses who reported taking no action, the two most common reasons given were that they “don’t know what to do” (23.6%), or they “do not want to think about it” (23.1%). Other reasons were: “I don’t think it will make a difference” (17.1%), “the expense factor” (14.4%), “I think the emergency services will help” (13.4%) and “I haven’t had time” (9.3%).

### 4.2 Nurses’ perceived and actual household preparedness

Three tests were performed to explore the extent to which the measures of perceived and actual household preparedness were interconnected. Firstly, chi-squared (*χ*^2^) tests of association were conducted between perceived preparedness and planning ($\chi^{2}=78.440\;df=10,p<0.001$), action ($\chi^{2}=43.193\;df=6,p<0.001$) and resources ($\chi^{2}=56.458\;df=6,p<0.001$).

The results in Table 5, the Cross-tabulation of perceived and actual household preparedness, indicate a significant association between perceived and actual preparedness. For example, 73.3% of nurses who perceived they were not prepared had less than three of the five items included in the Planning portion of the 24-item Household Preparedness Score (see Table 4), while 80.4% of nurses who felt they were prepared had three or more items.

**Table 5:** Cross-tabulation of perceived and actual preparedness.

	Actual Preparedness							
	Number of Planning Preparedness Items/Actions Taken							
Perceived Preparedness	**Planning**	[0]	[1]	[2]	[3]	[4]	[5]	n
	No	7 (9.9%)	27 (38.0%)	18 (25.4%)	11 (15.5%)	7 (9.9%)	1 (1.4%)	71
	Somewhat	15 (5.3%)	64 (22.7%)	70 (24.8%)	74 (26.2%)	49 (17.4%)	10 (3.5%)	282
	Yes	1 (1.3%)	3 (4.0%)	8 (10.7%)	20 (26.7%)	28 (37.3%)	15 (20.0%)	75

	**Action**	[0]	[1]	[2]	[3]			n
	No	11 (15.5%)	20 (28.2%)	31 (43.7%)	9 (12.7%)			71
	Somewhat	23 (8.5%)	59 (21.8%)	95 (35.1%)	94 (34.7%)			271
	Yes	2 (2.8%)	8 (11.1%)	16 (22.2%)	46 (63.9%)			72

	**Resources**	[1–4]	[5–8]	[9–12]	[13–16]			n
	No	11 (15.5%)	32 (45.1%)	26 (36.6%)	2 (2.8%)			71
	Somewhat	11 (3.9%)	99 (35.1%)	133 (47.2%)	39 (13.8%)			282
	Yes	1 (1.3%)	10 (13.3%)	38 (50.7%)	26 (34.7%)			75

Note: *p*<0.001

Secondly, Spearman’s correlations showed weak, but statistically significant, positive correlations between each of the four constructs for actual preparedness and perceived preparedness. Perceived preparedness correlated with action (*r* = 0.301, *p*<0.01), resources (*r* = 0.319, *p*<0.01), planning (*r* = 0.382, *p*<0.01) and Household Preparedness Score (*r* = 0.419, *p*<0.01). These results are in line with the findings of Kirschenbaum et al. [54] and Basolo et al. [55], who found perceived and actual preparedness were significantly, if weakly, correlated: r = .401, p < .01 and r = .18 to r = .33, p = .05 respectively.

To provide a comprehensive analysis of the relationship between perceived and actual preparedness, two ordered probit regression models were estimated (see Table 6, Ordered Probit Analysis with Marginal Effects). Perceived preparedness was chosen as the dependent variable in the regressions without implying a causal relationship exists.

**Table 6:** Ordered probit analysis with marginal effects.

	Dependent Variable: Perceived Preparedness				
Explanatory variables	Coef.	Robust Std. Err.	Marginal effects (dy/dx)		
Regression Model 1			No	Somewhat	Yes
Household Preparedness Score (Z-Score)	0.706***	0.084	-0.157***	-0.001	0.158***
Cut 1	-1.007	0.081			
Cut 2	1.154	0.082			
Wald Chi-squared (df = 1)	χ2 = 70.43***				
N	412				
**Regression Model 2**					
Planning Preparedness Scale (Z-Score)	0.222***	0.069	-0.055**	-0.0455**	0.097***
	0.468***	0.094			
Resources Preparedness Scale (Z-Score)	0.154*	0.078	-0.036	-0.004	0.032*
Action Preparedness Scale (Z-Score)	0.2005**	0.070	-0.0465**	-0.005	0.0415**
Cut 1	0.980	0.078			
Cut 2	-1.238	0.103			
Wald Chi-squared (df = 4)	χ2 = 71.59***				
N	412				

Note

* *p*<0.05

** *p<*0.01

*** *p*<0.001

For the ordered probit analysis, variables were tested for adherence to the parallel regressions assumption, using the auto fit procedure in the gologit2 command. For those independent variables which violate the parallel regressions assumption, coefficients are permitted to vary across the levels of the dependent variable, as shown in Eq 1, resulting in two coefficients reported in the tables below. Only one coefficient exists for those variables that satisfy the parallel regressions assumption.

Model 1 reports standard ordered probit results, as the parallel regressions assumption was not violated; that is, the chi-square test for equality of the independent variable coefficients was not rejected ($\chi_{1}^{2}=2.18,p=0.139$). In Model 2, the planning preparedness variable violated the parallel regressions assumption, while the other two independent variables did not.

The results in Table 6, Ordered Probit Analysis with Marginal Effects, show a significant positive relationship between actual preparedness and perceived preparedness. The marginal effects for Regression Model 1 show a one-point increase in the Household Preparedness Score increased the probability that nurses stated they were prepared for an emergency by 0.158 (i.e., a 15.8 percentage point (pp) change for Think-Yes). Regression Model 2 shows a significant positive effect of actual measures of planning, resources and action on perceived preparedness. Of the three factors used to measure preparedness, planning was shown to have the largest positive effect on nurses’ perceived preparedness, with the marginal impact of planning estimated as an increase of almost 0.1 (9.7 pp) in the probability nurses stated they are prepared (Think-Yes). These results confirmed that perceived and actual preparedness are not independent of one another among nurses.

### 4.3 Risk rating, exposure and preparedness

The results in Table 7, Regression Analysis of Perceived and Actual Preparedness, and Table 8, the marginal effects of the ordered probit model, illustrate the relationship between risk rating, prior exposure to emergencies, socio-demographic factors and the dependent variables, perceived and actual preparedness.

**Table 7:** Regression analysis of perceived and actual preparedness.

	Dependent Variable: Household Preparedness Score (Z-Score) †			Dependent Variable: Perceived Preparedness ‡		
Explanatory variables	Coef.	Robust Std. Err.	P	Coef.	Robust Std. Err.	P
Female	0.038	0.149	0.798	0.244	0.258	0.345
Age 35–44	0.035	0.117	0.767	-0.054	0.226	0.811
Age 45 & Older	-0.094	0.161	0.562	-0.444ǂ	0.259	0.086
Live–village	-0.193	0.139	0.165	-0.244	0.267	0.360
Live–town	-0.028	0.111	0.801	-0.397ǂ	0.229	0.083
				0.455*	0.214	0.034
Live–suburbs/outskirts of a city	-0.370**	0.127	0.004	-0.073	0.229	0.751
Live–city	-0.398**	0.147	0.007	-0.604*	0.246	0.014
Owns home	0.525****	0.110	0.001	0.418*	0.177	0.018
Years at address	0.003	0.006	0.655	0.010	0.009	0.304
Number of adults at household	0.059	0.046	0.204	0.019	0.082	0.813
Number of children (age <18) at household	-0.028	0.037	0.444	-0.179**	0.068	0.008
Income 30,000–70,000	0.465**	0.160	0.004	0.341	0.284	0.230
Income Over 70,000	0.674***	0.177	0.001	0.272	0.322	0.398
Years practicing	0.004	0.006	0.577	-0.002	0.011	0.832
Self-efficacy	0.045	0.035	0.196	0.121ǂ	0.064	0.058
Risk rating: socio-natural	-0.013	0.016	0.426	-0.029	0.029	0.310
Risk rating: technological	-0.012	0.019	0.530	-0.004	0.033	0.905
Risk rating: civil	0.009	0.016	0.573	-0.016	0.030	0.601
Exposure to socio-natural emergencies	0.080**	0.034	0.020	0.304***	0.083	0.001
				0.10	0.080	0.223
Exposure to technological emergencies	0.074	0.064	0.247	0.116	0.102	0.258
Exposure to civil emergencies	0.018	0.035	0.609	-0.119	0.080	0.137
				0.085	0.077	0.270
Constant	-0.868***	0.302	0.001			
Cut 1				0.918	0.546	
Cut 2				-1.303	0.558	
R^2^	.268					
F	5.22***					
Wald Chi-squared (df = 24)				χ2 = 72.69***		
N	322			298		

Notes

† OLS Regression

‡ Ordered Probit Regression

ǂ *p*≤0.1

* *p*<0.05

** p<0.01

*** *p*<0.001

**Table 8:** Marginal effects.

	Dependent Variable: Perceived Preparedness					
	Marginal Effects (dy/dx)					
	No	P	Somewhat	P	Yes	P
Female	-0.050	0.349	-0.006	0.576	0.056	0.346
Age 35–44	0.011	0.812	0.001	0.813	-0.012	0.811
Age 45 & Older	0.091ǂ	0.095	0.010	0.521	-0.101ǂ	0.088
Live–village	0.050	0.362	0.006	0.581	-0.056	0.359
Live–town	0.081ǂ	0.079	-0.185***	0.001	0.104*	0.035
Live–suburbs/outskirts of a city	0.015	0.751	0.002	0.771	-0.017	0.750
Live–city	0.124*	0.017	0.014	0.513	-0.138*	0.015
Owns home	-0.086*	0.018	-0.010	0.529	0.095*	0.022
Years at address	-0.002	0.309	0.000	0.558	0.002	0.302
Number of adults at household	-0.004	0.813	0.000	0.822	0.004	0.813
Number of children (age <18) at household	0.037**	0.008	0.004	0.520	-0.041**	0.009
Income 30,000–70,000	-0.070	0.235	-0.008	0.538	0.078	0.225
Income Over 70,000	-0.056	0.399	-0.006	0.586	0.062	0.394
Years practicing	0.000	0.832	0.000	0.840	-0.001	0.832
Self-efficacy	-0.025ǂ	0.065	0.003	0.519	0.028ǂ	0.060
Risk rating: socio-natural	0.006	0.310	0.001	0.573	-0.007	0.309
Risk rating: tech.	0.001	0.905	0.000	0.906	-0.001	0.905
Risk rating: civil	0.003	0.600	0.000	0.687	-0.004	0.602
Exposure to socio-natural emergencies	-0.062***	0.001	0.040ǂ	0.053	0.022	0.223
Exposure to tech. emergencies	-0.024	0.268	-0.003	0.551	0.026	0.261
Exposure to civil emergencies	0.024	0.137	-0.044*	0.028	0.019	0.269

Notes: Marginal effects are estimated using the mean values for all other explanatory variables

ǂ p≤0.1

*p<0.05

**p<0.01

***p<0.001

The regression analysis of perceived preparedness reveals four independent variables for which the parallel regression assumption was violated: living in a town, exposure to socio-natural emergencies, and exposure to civil emergencies. At a 5% significance level, the coefficients on these variables were found to vary over the latent perceived preparedness scale.

Overall, risk rating of socio-natural, technological, and civil risks was not significantly related to either perceived or actual preparedness. Regarding exposure, while prior exposure to technological and civil emergencies did not affect perceived or actual preparedness, the results indicate a significant positive relationship between exposure to socio-natural emergencies and both preparedness measures. This supported the findings from the literature review, which suggested exposure to some types of emergencies would have a significant positive effect on household preparedness [55, 60, 65, 73, 81, 90, 92, 93, 121–123]. The results obtained from the generalized ordered probit model revealed a more nuanced influence between exposure to socio-natural emergencies and perceived preparedness, suggesting exposure to socio-natural emergencies had a significant impact on perceived preparedness only at the lower levels of the scale. The associated marginal effects show exposure to socio-natural emergencies leads to an estimated decrease of 6.2pp (p ≤0.001) in the probability that a nurse stated they were not prepared (Think-No) and increased the probability a nurse stated they are somewhat prepared (Think-Somewhat) by 4pp (p = 0.053). There is no significant impact on the higher level of perceived preparedness (Think-Yes).

Gender, years of residence, age and the number of adults in the household were not significant in either the perceived or actual preparedness models. Conversely, homeownership and living in a city were significant in both models. Confirming prior studies, this study found homeownership presented as a positive predictor of nurses having higher levels of actual (see [55, 56, 81]) and perceived (see [61, 65]) preparedness, while living in a city, rather than a rural setting, was found to be a negative predictor of both. As previously noted, the studies on perceived and actual preparedness have not examined this urban/rural divide.

Nurses who had children were less likely to consider themselves prepared. The marginal effects showed that for each additional child in the household, the probability that nurses felt prepared (Think-Yes) decreased by an estimated 4.6pp (p ≤0.01). This finding differed from previous studies of general households, which suggested that the relationship between children’s presence in the home and perceived preparedness was non-significant [55, 56, 61, 68]. The results in Table 7 also show that living in a town leads to greater polarization of opinion on preparedness than living in a rural setting; i.e., those living in a town are less likely to perceive themselves as somewhat prepared and more likely to state either a definite yes or no. Similarly, nurses’ self-efficacy was significant only in the context of perceived preparedness, with a higher likelihood of providing a definitive answer, either "yes" or "no". The related marginal effects demonstrated that for each increment in self-efficacy, the likelihood of nurses feeling prepared (categorized as "Think-Yes") rose by an estimated 2.8 percentage points (p = 0.06). Nurses aged 45 and older, compared to 34 or under, were less likely to indicate higher levels of perceived preparedness. The associated marginal effects show that nurses aged 45 and older were less likely to state they were prepared (Think-Yes) by 10.1pp (p ≤0.1). The remainder of the factors for perceived preparedness were non-significant. For actual preparedness, in addition to the previously mentioned relationship to urban/rural factors, there is evidence of a positive relationship with household income. This finding was in line with some studies on preparedness [59, 73, 92, 123]. However, others had suggested a non-significant effect [55, 56, 102, 124].

## 5. Discussion and recommendations

As evident from the COVID-19 pandemic, frontline health workers accomplish indispensable work in the face of “added clinical activity and increased demands on the workforce and available resources” [96]. As well as being professionally prepared, there is an expectation that outside the clinical setting they are personally prepared in advance of an emergency [1, 2].

The literature review revealed only a limited number of studies examined the influence of risk rating, prior exposure to emergences, and socio-demographic characteristics on both perceived and actual household preparedness within a single study [53–56]. Moreover, none of these studies had examined frontline healthcare personnel. Therefore, with prior research showing that a lack of household preparedness can become a barrier to healthcare staff reporting to work during an emergency [12, 26, 56, 57], this study investigated nurses’ household emergency preparedness.

A key finding of this study was that perceived and actual household preparedness were not independent of each other among nurses. The marginal effect estimations showed that a one-point increase in the Household Preparedness Score led to a 15.8pp rise in the probability that a nurse feels prepared. This indicates that nurses associate their perception of preparedness with the protective actions taken. This connection between perceived and actual household preparedness is critical if nurses are to feel confident to leave their homes and families and report for duty during an emergency.

To maximize attendance during an emergency, healthcare management should train nurses on ways to prepare their households for an emergency. Evidence from the literature review suggests household preparedness campaigns are the most effective way to promote preparedness [119]. Campaigns such as the “Ready Responder Toolkit” by FEMA [5] could be used to raise nurses’ actual household emergency preparedness. These campaigns could include checklists of preparedness items/actions, such as the list given in Table 4, and remind nurses to keep a list of emergency contact numbers (which 52.1% of the sample did not have), enough water for 3+ days (absent in 59.7% of nurses’ homes), and enough medication for 8+ days (not present in 54.4% of nurses’ homes). However, building actual preparedness is only one dimension. It’s equally crucial that nurses feel confident in their ability to handle emergencies. Even with the most effective training and guidance, if nurses do not believe they are prepared, it could affect their performance and decision-making during an emergency. As such, once actual preparedness has been built, a more psychosocial approach should be adopted to help ensure personnel feel prepared. After nurses have been trained and have taken necessary protective actions, healthcare management could engage in activities that boost morale, confidence, and the internal belief of being ready. This could include debriefing sessions where nurses can discuss their feelings in post-training workshops that address emergency-related anxieties.

Providing checklists of preparedness items/actions and encouraging the need to evaluate household preparedness against the checklist, for example in a workshop, should improve household preparedness and assist in the alignment of perceived and actual preparedness (Fig 1: Think-Yes & Actual-Yes). Without this two-pronged approach, as noted by Kohn et al. [115] and Martin [29], a lack of preparedness could negatively impact the capacity and functioning of a healthcare system during an emergency.

In addition to establishing that none of the previous studies examining both perceived and actual household preparedness had studied a sample of nurses, the literature review revealed inconsistent results between studies on the relationship between perceived and actual preparedness and socio-demographic factors. The design of this study allowed us to test for differences in the factors influencing perceived and actual preparedness using one sample. The findings support the contention that differences exist.

A deeper understanding of the factors which influence perceived and actual preparedness should assist with the promotion of both perceived and actual preparedness. The analysis showed that homeownership (positive) and living in a city (negative) were the only two significant socio-demographic variables in both models. Additionally, exposure to socio-natural emergencies was positive and significant in both preparedness measures. The impact of exposure to socio-natural emergencies did not, however, extend to the higher levels of perceived preparedness. To maximize effectiveness, the household preparedness campaign should target information towards specific demographics. For example, campaigns focused on building perceived and actual preparedness should target nurses who live in urban areas and those who rent their homes. The analysis showed that nurses with children had lower perceived preparedness, while nurses in lower-income roles had lower actual preparedness. From an emergency preparedness perspective, it would seem wise that both cohorts are supported to avoid them feeling, or being, underprepared at home. As the factors influencing actual preparedness were not always the same as those influencing perceived preparedness, preparedness campaigns should be designed with both perceived and actual preparedness in mind.

### 5.1 Limitations

Though the data, collected in 2016, might be perceived as having dated over the past few years, it’s crucial to highlight that in the subsequent seven years, there have been no initiatives to enhance the household preparedness of nurses or other frontline responders. Furthermore, hospitals continue to grapple with overcapacity, staff burnout, and overwhelming workloads [125]. Consequently, the operational setting of this research has remained largely consistent in Ireland.

As noted within the methodology, respondents reported perceived preparedness before actual preparedness. This was seen as vital to mitigate the effects of common method bias by having respondents provide an instinctive and intuitive assessment of their household preparedness before being shown a list of recommended measures that could alter the assessment. That said, this method is not without limitations, we do not know on what information respondents drew to answer this question nor if they provided the answer which they thought we wanted to hear (social desirability bias). Future studies could mitigate these limitations by gathering data using an interview methodology and exploring the answers in greater depth.

Actual preparedness was measured using a set of items/actions that are in line with national and international preparedness guidelines. However, this list does not account for everything a household may have which could help them cope in an emergency. Furthermore, there is a reliance on an accurate assessment by the respondent. A household audit would close this gap but would limit the scope of the study and may pose challenges when gaining ethical approval.

The independent variables included within the regression models do not account for all the factors which may influence perceived and actual preparedness. For example, protection motivation variables such as perceived self-efficacy, response cost, and worry could be included in future models.

This study was restricted to Ireland, limiting the generalizability of the findings to nurses in other countries. To address this and build confidence in our results, we suggest replicating the study in other countries.

As this marks Ireland’s first in-depth study into nurses’ household preparedness, our study acts as a baseline for future research. This will allow for comparisons to be made, especially as new preparedness strategies are rolled out to enhance household preparedness for nurses in Ireland.

## 6. Conclusion

This research used an online questionnaire to assess nurses perceived and actual household preparedness while controlling for factors such as, risk rating, exposure to prior emergencies, and self-efficacy. Perceived preparedness was evaluated based on each nurse’s subjective feeling of preparedness for potential emergencies affecting their home. Actual preparedness, on the other hand, was quantified through an assessment of 24 specific preparedness measures and actions undertaken within their households, including the development of an emergency plan and maintaining a sufficient food supply for more than three days.

The research questions and the findings linked to each of these are reported in the Table 9 below.

**Table 9:** Key findings.

Hypothesised Outcomes Per the Literature Review	Key Findings
Research Question 1: How aligned are nurses’ perceived and actual household preparedness	
Hypothesis 1: There is a significant positive alignment between nurses’ perceived household preparedness and their actual household preparedness.	Hypothesis confirmed.
	Results showed a positive relationship between nurses’ perceived household preparedness and their actual household preparedness
Research Question 2: To what extent do risk rating, prior exposure to emergencies, and socio-demographic factors influence perceived and actual preparedness among nurses?	
Hypothesis 2: Risk rating is positively and significantly associated with higher levels of perceived and actual preparedness. High risk ratings suggest awareness of risks and potential vulnerability, which should prompt preparedness action.	Hypothesis rejected.
	High Risk ratings for socio-natural, technological, and civil risks were not significantly related to perceived or actual preparedness.
Hypothesis 3: If a respondent has undertaken the identified preparedness measures, this may result in a lower assessment of risk [106]. If this is the case, we may see low levels of risk rating associated with high levels of perceived preparedness. This could lead to nurses not reviewing or revisiting their household preparedness or they may ignore public awareness campaigns designed to drive household resilience.	Hypothesis rejected.
	Low Risk ratings for socio-natural, technological, and civil risks were not significantly related to either perceived or actual preparedness.
Hypothesis 4: Exposure to an emergency is associated with higher levels of actual preparedness as experiencing an emergency leads to the taking of measures to increase household preparedness.	Hypothesis confirmed.
	Testing confirmed a significant positive relationship between exposure to socio-natural emergencies and actual preparedness.
	Prior exposure to technological and civil emergencies did not affect actual preparedness,
Hypothesis 5: direct exposure to emergencies may lead to a non-protective response (denial, fatalism) and feelings that it is not possible to protect against future emergencies. In such circumstances exposure to an emergency is associated with lower levels of perceived preparedness.	Hypothesis rejected.
	Results showed exposure to socio-natural emergencies had a significant positive impact on perceived preparedness only at the lower levels of the scale. There was no significant impact on the higher level of perceived preparedness.
	Prior exposure to technological and civil emergencies did not affect actual preparedness.
Hypothesis 6: Socio-demographic factors significantly impact both perceived and actual preparedness among nurses.	Hypothesis confirmed.
	Results confirmed homeownership and living in a city significantly impacted both perceived and actual preparedness among nurses.

This deeper understanding of the factors that influence perceived and actual household emergency preparedness among nurses is required to facilitate higher levels of emergency preparedness in healthcare facilities. Within the Irish context, the Nursing and Midwifery Board of Ireland could collaborate with the Be Winter and Be Summer Ready Campaigns [104] to initiate a nationwide campaign emphasizing the emergency preparedness of nurses’ households. Integrating with a national campaign would reduce expenses for the health sector and promote a consistent strategy across all hospitals. Aligning with Trainor and Barsky’s [11] suggestion, special rates on preparedness items and measures could be arranged for nurses to help alleviate the cost of household preparedness. Ensuring nurses actual household preparedness and perceived preparedness are aligned and high means that they are prepared, and feel prepared, for an emergency. Preparedness actions, such as those listed in Household Preparedness Score, should ease concerns and allow nurses to concentrate on their professional responsibilities during an emergency.

## Supporting information

S1 Checklist *PLOS ONE* clinical studies checklist. (DOCX)

S1 Table Summary of literature review. (DOCX)

S2 Table Summary of the studies that examined perceived and actual preparedness using regression analysis. (DOCX)

S3 Table S3.1 Table regression analysis of perceived and actual preparedness & S3.2 Table marginal effects. (DOCX)
