# Supplementary material for: An assessment of nurses’ perceived and actual household emergency preparedness
Source: PLoS One. 2024 Apr 18;19(4):e0300536. doi: 10.1371/journal.pone.0300536 (PMC11025835; doi:10.1371/journal.pone.0300536)
Supplement: S1 Table — (DOCX) [file pone.0300536.s002.docx]

**An assessment of nurses’ perceived and actual household emergency preparedness**

Gavin David Brown^1 *^

Caroline McMullan ^1^

Ann Largey ^1^

David Leon ^1^

* Corresponding Author

E-mail address: [gavin.brown@dcu.ie](mailto:gavin.brown@dcu.ie)

**S1 Table Summary of literature review**

Notes: +ve = Positive and significant relationship, -ve = negative and significant relationship, NS = non-significant.

| **Ref** | **Disaster** | **Preparedness Variable** | **Model** | **Analysis** | **Female** | **Age** | **Owns Home** | **Years of residence** | **Urban (Inc. Town)** | **Education** | **Children** | **Income** | **Race** | **Marital Status** | **Household Size** | **Employed** | **Disaster Exposure** | **Risk Rating / Risk Perception** | **Other Factors** | |
| --- | --- | --- | --- | --- | --- | --- | --- | --- | --- | --- | --- | --- | --- | --- | --- | --- | --- | --- | --- | --- |
| ^1^ | Earthquake | Actual preparedness | Actual | Regression | NS | NS | - | - | - | +ve | - | - | - | - | - | - | +ve | - | Living in risk zone, NS  Religion, -ve  Trust in information source, NS  Past experience*trust, NS | |
|  |  | Actual preparedness for Israeli Jews | Actual | Regression | NS | NS | - | - | - | NS | - | - | - | - | - | - | +ve | - | Living in risk zone, NS  Trust in information source, NS  Past experience*trust, -ev | |
|  |  | Actual preparedness for Israeli Arabs | Actual | Regression | NS | NS | - | - | - | +ve | - | - | - | - | - | - | NS | - | Living in risk zone, NS  Trust in information source, NS  Past experience*trust, +ev | |
|  |  | Perceived preparedness | Perceived | Regression | +ve | -ve | - | - | - | NS | - | - | - | - | - | - | NS | - | Living in a risk zone, +ve Religion, NS Trust in information source, NS  Past experience*trust, NS | |
|  |  | Perceived preparedness for Israeli Jews | Perceived | Regression | +ve | -ve | - | - | - | NS | - | - | - | - | - | - | NS | - | Living in risk zone, NS  Trust in information source, NS  Past experience*trust, NS | |
|  |  | Perceived preparedness for Israeli Arabs | Perceived | Regression | NS | -ve | - | - | - | NS | - | - | - | - | - | - | NS | - | Living in risk zone, +ev  Trust in information source, NS  Past experience*trust, NS | |
| ^2^ | Earthquakes / Hurricane | Perceived level of preparedness for earthquakes | Perceived | Logit | NS | NS | NS | - | - | NS | NS | NS | NS | NS | - | - | NS | >  See next column | Risk Rating / Risk Perception  Dread, NS Fatal, NS Happen, NS  Other Factors  Confidence in government, +ve Information sources, +ve | |
|  |  | Perceived level of preparedness for hurricanes | Perceived | Logit | NS | NS | NS | - | - | NS | NS | NS | NS | NS | - | - | NS | >  See next column | Risk Rating / Risk Perception  Dread, NS Fatal, -ve Happen, NS  Other Factors  Confidence in government, +ve Information sources, +ve | |
|  | Earthquakes | Preparedness actions for earthquakes - family plan | Actual | Logit | NS | NS | NS | - | - | NS | NS | NS | NS | NS | - | - | NS | >  See next column | Risk Rating / Risk Perception  Dread, NS Fatal, NS Happen, NS  Other Factors  Confidence in government, NS Information sources, +ve | |
|  |  | Preparedness actions for earthquakes - supplies | Actual | Logit | NS | NS | NS | - | - | NS | NS | NS | NS | NS | - | - | NS | >  See next column | Risk Rating / Risk Perception  Dread, NS Fatal, NS Happen, NS  Other Factors  Confidence in government, NS Information sources, NS | |
|  |  | Preparedness actions for earthquakes - mitigation | Actual | Logit | NS | NS | NS | - | - | NS | NS | NS | NS | NS | - | - | NS | >  See next column | Risk Rating / Risk Perception  Dread, NS Fatal, NS Happen, NS  Other Factors  Confidence in government, NS Information sources, NS | |
|  |  | Preparedness actions for earthquakes - shut off utilities | Actual | Logit | NS | NS | +ve | - | - | NS | NS | NS | NS | NS | - | - | NS | >  See next column | Risk Rating / Risk Perception  Dread, NS Fatal, NS Happen, NS  Other Factors  Confidence in government, NS Information sources, +ve | |
|  | Hurricane | Preparedness actions for hurricanes - family plan | Actual | Logit | NS | NS | NS | - | - | NS | NS | NS | NS | +ve | - | - | NS | >  See next column | Risk Rating / Risk Perception  Dread, +ve Fatal, NS Happen, NS  Other Factors  Confidence in government, +ve Information sources, NS | |
|  |  | Preparedness actions for hurricanes - supplies | Actual | Logit | NS | NS | +ve | - | - | NS | +ve | NS | NS | NS | - | - | NS | >  See next column | Risk Rating / Risk Perception  Dread, NS Fatal, NS Happen, NS  Other Factors  Confidence in government, NS Information sources, NS | |
|  |  | Preparedness actions for hurricanes - shut off utilities | Actual | Logits | -ve | +ve | NS | - | - | NS | NS | NS | NS | NS | - | - | NS | >  See next column | Risk Rating / Risk Perception  Dread, NS Fatal, NS Happen, NS  Other Factors  Confidence in government, NS Information sources, NS | |
| ^3^ | Hurricane | Perceived level of preparedness | Perceived | Ordered Logit | NS | +ve | NS | - | - | NS | NS | NS | NS | NS | - | - | NS | +ve | Immigrant, -ve Information, NS Perceived readiness of local government, +ve | |
|  |  | House preparedness (sandbags on site) | Actual | Logit | -ve | NS | NS | - | - | NS | +ve | NS | -ve | +ve | - | - | NS | +ve | Immigrant, NS Information, NS Perceived readiness of local government, NS | |
|  |  | Household preparedness (family plan) | Actual | Logit | NS | NS | NS | - | - | NS | NS | NS | NS | NS | - | - | +ve | NS | Information (a lot), +ve Perceived readiness of local government, +ve | |
|  |  | Preparedness (all recommended items on hand) | Actual | Logit | NS | NS | +ve | - | - | NS | NS | NS | NS | +ve | - | - | NS | NS | Immigrant, NS Information (a lot), NS Perceived readiness of local government, NS | |
| ^4^ | Volcanic | Composite score for preparedness  -Planning Score -Supplies Score -Action Score | Actual | T-tests | - | - | - | - | - | - | - | - | - | - | - | - | - | - | A statistically significant difference in preparedness exists for five of the 20 preparedness measures listed.  Please see original paper for list. | |
| ^5^ | Multi-Hazard Context | Preparedness | Actual | Summary statistics | - | - | - | - | - | - | - | - | - | - | - | - | - | - | Please see paper for full results.  Responder and family preparedness - finding listed. 65.9% of organisations provide general emergency preparedness training and education opportunities for employees. | |
| ^6^ | Terrorism | Preparedness | Actual | Path Analysis | - | - | - | - | - | - | - | - | - | - | - | - | - | NS | Direct paths only  Risk Rating / Risk Perception  Note: Perceived risk = Likelihood  Other Factors  Knowledge, +ve Response efficacy, +ve Milling behaviour, +ve | |
| ^7^ | Natural Hazard - Multi-Hazard Context | Household perceived preparedness | Perceived | Regression | NS | - | NS | NS | - | - | - | - | - | - | - | - | - | - | Know how to fish, NS Know how to preserve food, NS Origin: Mainland, NS Trade fish, NS The profession - Teacher, +ve Have a safe place to evacuate to, NS Know of safe places to evacuate to, NS Know of public awareness programs, NS Think early warning systems are very effective, NS | |
|  |  |  |  |  | NS | NS | - | -ve | - | - | - | - | - | - | - | - | NS | - | The profession - Teacher, NS Have a safe place to evacuate to, +ve Participation in a Community Group, NS | |
|  |  |  |  |  | NS | NS | - | -ve | - | - | - | NS | - | - | - | - | +ve | - | Have a safe place to evacuate to, NS | |
|  |  |  |  |  | NS | NS | - | -ve | - | - | - | - | NS | - | - | - | - | - | Know of public awareness programs, +ve Think early warning systems are somewhat  effective, NS | |
|  |  | Coping capacity (e.g. stocked food/water supplies/emergency rations) | Actual | Regression | NS | - | - | -ve | - | NS | - | NS | - | - | - | - | - | - | Perceived as Prepared, NS Feel the community is prepared, NS Origin, NS Participation in a Community Group, +ve | |
|  |  |  |  |  | NS | - | - | NS | - | - | - | - | - | - | - | - | - | - | Perceived as Prepared, NS Feel the community is prepared, +ve Origin, NS | |
| ^8^ | Natural Hazard "environmental threat (coastal)" | Perceived preparedness | Perceived | Logit | NS | -ve | +ve | - | - | -ve | NS | NS | NS | NS | NS | -ve | - | +ve | Model 1  Risk Rating / Risk Perception  Note: At-risk (risk perception)  Other Factors  Local resources (social capital), NS Extra-local resources (social capital), +ve Regional resources (social capital), +ve Community associations (social capital), NS General trust (social capital), NS Number of adults know - name (social capital), NS Length of residence (social capital), NS Work in the oil industry, -ve Work in the fishing industry, NS | |
| ^9^ | Flooding | Perceptions of preparedness | Perceived | Linear Regression | - | - | - | - | - | - | - | - | - | - | - | - | - | >  See next column | Risk Rating / Risk Perception  Susceptibility, NS  Certainty, +ve Immediacy, +ve  Other Factors  Response-efficacy, -ve Self-efficacy, +ve Salience (perceived threat salience), NS | |
| ^10^ | Flooding | Preparedness | Perceived | T-test | -ve | - | - | - | - | - | - | - | - | - | - | - | +ve | - | Hazard Awareness, NS Worry, NS Evacuated properties in the previous flood, +ve | |
| ^11^ | Public Health Emergencies | Level of perceived preparedness | Perceived | Logit | -ve | +ve | - | - | - | -ve | - | - | +ve | - | - | - | - | - | Survey language, English, +ve | |
|  |  | Number of preparedness items | Actual | Logit | -ve | +ve | - | - | - | -ve | - | - | -ve | - | - | - | - | - | (5-Items)  Survey language, English, +ve | |
| ^12^ | Disaster | Preparedness (self-assessment) | Perceived | Ordered Logit | NS | NS | +ve | - | - | NS | - | - | +ve | NS | - | - | +ve | >  See next column | Risk Rating / Risk Perception  Likelihood, NS  Consequence, +ve  Other Factors  Average loss, NS  Risk tolerance, +ve Self-reliance, +ve Future orientation, NS  Compliance, +ve Informed, +ve | |
| ^13^ | Earthquake | Perceived preparedness | Perceived | Multivariate Regression | - | - | - | - | - | - | - | - | - | - | - | NS | NS | - | Trust, +ve  Residence region, NS Family members, -ve | |
|  |  | Actual preparedness behaviour | Actual | Multivariate Regression | -ve | NS | NS | - | - | - | - | - | - | - | - | - | NS | - | Trust, +ve  Family members, +ve Monthly income, +ve | |
| ^14^ | Climate Change | Perceived capacity to prepare | Perceived | Linear Regression | NS | NS | - | - | - | - | - | +ve | - | - | -ve | - | - | - | (Prepare: 4) Household head, NS Expenditure, +ve Exp*income = 1, -ve Exp*income = 2, NS | Exp*income = 3, NS Friends (network), NS Relatives (Network), +ve NGO (Network), NS Government, NS |
| ^15^ | Disasters | Perceived preparedness | Perceived | Multivariate Logits | NS | +ve | - | - | - | +ve | NS | NS | NS | - | - | - | - | - | US census region (South), +ve Metropolitan statistical area, NS | |
| ^16^ | Earthquakes | Preparedness | Perceived | Ordinal Logit | - | - | - | - | - | - | - | - | - | - | - | - | - | +ve | Impact, +ve  Family trust, NS General trust, NS Specific trust, NS Government trust -ve, Support, +ve | |
| ^17^ | Disasters | 72-hour disaster kit | Actual | Logit | -ve | - | - | - | - | +ve | NS | +ve | - | +ve | - | - | +ve | - | Duty Status, +ve (Veterans) Deployed Member, NS Chronic Health Condition, +ve Will, +ve Disaster Kit for Two Weeks, +ve Safe Shelter, +ve Secondary Shelter, +ve Emergency Plan, +ve Communication Plan, +ve Evacuation Plan, +ve Taken a Preparatory Class, +ve Live in Disaster Area, +ve | |
| ^18^ | Fire and Medical | Fire extinguisher | Actual | Logit | NS | NS | +ve | - | - | NS | +ve | - | - | NS | +ve | - | NS | NS | Note: Risk Perception = risk likelihood  Civil Defence/ Medical Sector, NS | |
|  |  | Fire blanket | Actual | Logit | -ve | NS | +ve | - | - | NS | NS | - | - | NS | +ve | - | NS | NS | Note: Risk Perception = risk likelihood Civil Defence/ Medical Sector, NS | |
|  |  | Smoke detectors | Actual | Logit | NS | NS | +ve | - | - | NS | NS | - | - | NS | +ve | - | NS | NS | Note: Risk Perception = risk likelihood Civil Defence/ Medical Sector, NS | |
|  |  | First aid kit | Actual | Logit | NS | +ve | +ve | - | - | NS | +ve | - | - | NS | +ve | - | NS | NS | Note: Risk Perception = risk likelihood  Civil Defence/ Medical Sector, NS | |
| ^19^ | Hurricane | Emergency contact | Actual | Logit | - | - | NS | - | - | - | NS | - | - | +ve | NS | - | NS | NS | Mobile home, NS Agreement, +ve | |
|  |  | Insurance | Actual | Logit | - | - | -ve | - | - | - | NS | - | - | +ve | NS | - | NS | NS | Mobile home, NS Agreement, -ve | |
|  |  | Evacuation zone | Actual | Logit | - | - | NS | - | - | - | NS | - | - | +ve | NS | - | NS | NS | Mobile home, NS Agreement, +ve | |
| ^20^ | Flooding | Preparedness scale | Actual | Tobit model | NS | - | - | - | - | - | - | - | - | NS | - | - | - | >  See next column | Risk Rating / Risk Perception  Risk Perception (likelihood), NS  Risk perception (feeling of worry), NS  Other Factors  Critical awareness (think about flood), NS Critical awareness (talk about flood), NS Country, -ve Sense of community, +ve Self-efficacy, -ve Responsibility efficacy, +ve Outcome expectancy, +ve Size of land, +ve Land ownership, NS Access to land, +ve | |
| ^21^ | Earthquake | Earthquake readiness scale | Actual | multiple linear Regression | NS | NS | -ve | - | - | NS | - | - | - | - | - | NS | -ve | - | Who the person lives with, NS | |
| ^22^ | Earthquake | Post-earthquake activities - some 1–5 vs. no preparation | Actual | Multinomial Logit | - | - | - | - | - | - | - | - | - | +ve | - | - | - | - | Physical injury, +ve Emotional injury, +ve Financial injury, NS  Immigrant status, NS  8-item scale of survival preparedness, +ve  4-item scale of hazard mitigation, NS  Perceived pre-quake preparedness, NS MMI (Modified Mercalli intensity), +ve Interaction: Financial injury & mitigation, +ve Interaction: Survival preparedness & hazard mitigation, -ve  Interaction: Survival preparedness and preparedness feelings, -ve | |
|  |  | Post-earthquake activities - many 6 or more vs. no preparation | Actual | Multinomial Logit | - | - | - | - | - | - | - | - | - | +ve | - | - | - | - | Physical injury, +ve Emotional injury, +ve Financial injury, +ve Immigrant status, +ve  8-item scale of survival preparedness, +ve  4-item scale of hazard mitigation, NS  Perceived pre-quake preparedness, NS  MMI (Modified Mercalli intensity), +ve Interaction: Financial injury & mitigation, +ve  Interaction: Survival preparedness & hazard mitigation, -ve  Interaction: Survival preparedness and preparedness feelings, -ve | |
| ^23^ | War (Armed Conflict) | Preparedness index (preparedness behaviour) | Actual | Linear Analysis | -ve | +ve | - | - | - | - | - | - | - | - | - | - | - | - | Sense of preparedness, +ve  Ignoring instructions, -ve | |
| ^24^ | Flooding | Household preparedness | Actual | Structural Equation Model | - | - | - | - | - | - | - | - | - | - | - | - | - | NS | Table 5: Household Preparedness Community participation, NS Collective efficacy, NS Perceived susceptibility, NS Perceived severity, NS Cues to action, -ve Perceived benefits minus barriers, -ve | |
| ^25^ | Flooding (Typhoons & Heavy Rains) | Disaster preparedness behaviours | Actual | Regression | - | - | - | - | - | - | - | - | - | - | - | - | +ve | +ve | Community Disaster Preparedness, +ve | |
| ^26^ | Disaster | Preparedness | Actual | Regression | - | - | - | - | - | - | - | - | - | - | - | - | - | +ve | Financial coping, +ve Trust in local government to respond, +ve Confidence for engaging in preparedness, +ve | |
| ^27^ | Natural Hazards (Typhoon & Earthquake) | Degree of preparedness | Actual | Poisson Regression | NS | NS | NS | - | -ve | +ve | NS | NS | - | NS | - | NS | >  See next column | | Disaster Exposure  Typhoon, +ve  Earthquake, NS  Both, NS  Risk Rating / Risk Perception  Typhoon probability, +ve  Typhoon consequence, -ve  Earthquake probability, +ve  Earthquake consequence,  -ve  Typhoon anxiety, +ve Earthquake anxiety, +ve | Other Factors Confidence, +ve Membership, +ve Daily contact, +ve Neighbour, +ve Residence, -ve status (Social), NS Religion, NS |
| ^28^ | Terrorism | Preparedness actions taken | Actual | Path analysis | - | - | - | - | - | - | - | - | - | - | - | - | - | - | Paths to Preparedness Actions taken  Preparedness Information Received, +ve Density of Preparedness Information Received, NS Preparedness Action Information Observed, +ve Knowledge of Preparedness Actions, +ve Perceived Effectiveness of Preparedness, +ve Milling About Preparedness Actions, +ve | |
| ^29^ | War (Armed Conflict) | Preparedness index (preparedness behaviour) | Actual | Multi-variant linear Regression | NS | - | - | - | - | - | - | - | - | - | - | - | - | -ve | Risk Rating / Risk Perception  Note: Severity of impact on family’s routine  Other Factors  Place of birth, +ve Place of residence, +ve Willingness to search for information, +ve Sense of preparedness, +ve | |
| ^30^ | Disasters (hurricanes, tornados, severe thunder-storms, and ice storms) | Disaster preparedness | Actual | Regression | - | - | - | - | - | - | - | - | - | - | - | - | - | +ve | Psychological sense of community, +ve Sense of place, -ve Preparedness self-efficacy, +ve Confidence in government, NS | |
| ^31^ | Earthquake | Disaster preparedness | Actual | Multiple Regression | - | - | - | - | - | - | - | - | - | - | - | - | - | - | Hazard and vulnerability awareness +ve Fatalistic beliefs -ve Denial beliefs -ve | |
| ^32^ | "Natural disasters" and Pandemics | Personal preparedness: disaster | Actual | Linear Regression | - | - | - | - | - | - | +ve | - | - | - | - | - | - | - | Perception of personal preparedness - Natural Disaster, +ve  Perception of personal preparedness - Pandemic, +ve  Years working in HR, +ve Employer encouragement to have a plan, +ve Disaster preparedness training in the last 2 years, +ve | |
|  |  | Personal preparedness: pandemic | Actual | Linear Regression | -ve | - | - | - | - | - | +ve | - | - | - | - | - | - | - | Perception of personal preparedness - Natural Disaster, +ve  Perception of personal preparedness - Pandemic, +ve | |
| ^33^ | Landslide | Household prepared some necessary items | Actual | Logit Model | NS | NS | - | - | - | NS | NS | NS | - | - | NS | - | NS | >  See next column | Risk Rating / Risk Perception  Probability, +ve  Threat, NS  Worry, -ve  Unknown, +ve  Controllability, NS | Other Factors  Place dependence, +ve  Place identity, NS  Place attachment, NS  Financial help, NS  Loss, NS  Distance, +ve  Official information, NS  All information, NS  Old, NS  Housing material, NS |
|  |  | Household learned knowledge of disaster prevention and mitigation | Actual | Logit Model | NS | NS | - | - | - | +ve | NS | NS | - | - | NS | - | NS | >  See next column | Risk Rating / Risk Perception  Probability, +ve  Threat, +ve  Worry, NS  Unknown, -ve  Controllability, -ve | Other Factors  Place dependence, +ve  Place identity, NS  Place attachment, NS  Financial help, NS  Loss, +ve  Distance, +ve  Official information, +ve  All information, NS  Old, NS  Housing material, -ve |
|  |  | Household participated in government organized disaster-related training and drills | Actual | Logit Model | NS | NS | - | - | - | +ve | NS | NS | - | - | NS | - | NS | >  See next column | Risk Rating / Risk Perception  Probability, +ve  Threat, NS  Worry, NS  Unknown, NS  Controllability, NS | Other Factors  Place dependence, NS  Place identity, NS  Place attachment, NS  Financial help, NS  Loss, NS  Distance, NS  Official information, NS  All information, NS  Old, NS  Housing material, NS |
|  |  | Household made some changes to home | Actual | Logit Model | NS | NS | - | - | - | NS | NS | -ve | - | - | +ve | - | +ve | >  See next column | Risk Rating / Risk Perception  Probability, +ve  Threat, NS  Worry, NS  Unknown, NS  Controllability, NS | Other Factors  Place dependence, NS  Place identity, NS  Place attachment, NS  Financial help, NS  Loss, NS  Distance, NS  Official information, +ve  All information, +ve  Old, -ve  Housing material, +ve |
|  |  | Household purchased any kind of insurance against natural hazard-induced disasters | Actual | Logit Model | +ve | NS | - | - | - | NS | +ve | +ve | - | - | -ve | - | NS | >  See next column | Risk Rating / Risk Perception  Probability, NS  Threat, +ve  Worry, NS  Unknown, +ve  Controllability, -ve | Other Factors  Place dependence, NS  Place identity, NS  Place attachment, +ve Financial help, -ve Loss, NS Distance, NS  Official information, +ve All information, NS Old, NS Housing material, NS |
|  |  | Total number of adopted disaster preparedness behaviours | Actual | Tobit mode | NS | NS | - | - | - | +ve | NS | NS | - | - | NS | - | +ve | >  See next column | Risk Rating / Risk Perception  Probability, +ve  Threat, +ve  Worry, NS  Unknown, NS Controllability, -ve | Other Factors  Place dependence, +ve Place identity, NS  Place attachment, NS Financial help, NS Loss, NS Distance, +ve  Official information, +ve All information, +ve Old, NS Housing material, NS |
| ^34^ | "Natural disaster" | Disaster preparedness - Philippines | Actual | Logit | - | NS | - | +ve | - | +ve | +ve | - | - | NS | -ve | NS | +ve | - | Health status, NS Mother has a secondary education, NS  Father has a secondary education, NS Started working at age <12, -ve % older people (aged >65) in hh, NS  % with secondary education in hh, NS  House located near a river, NS  House located near a mountain, -ve Area, +ve | |
|  |  | No. of measures (preparedness) - Philippines | Actual | Ordered Logit | - | NS | - | NS | - | +ve | +ve | - | - | NS | -ve | NS | +ve | - | Health status, +ve Mother has a secondary education, NS  Father has a secondary education, +ve Started working at age <12, NS % older people (aged >65) in hh, NS  % with secondary education in hh, NS  House located near a river, NS  House located near a mountain, NS Area, +ve | |
|  |  | Disaster preparedness - Thailand | Actual | Logit | NS | +ve | NS | NS | - | +ve | NS | - | - | NS | NS | NS | +ve | - | Health status, NS % older people (aged >65) in hh, NS  % with secondary education in hh, NS  Own land, NS House located near the coast, +ve House located near a river, NS  House located near a mountain, NS Area, -ve | |
|  |  | No. of measures (preparedness) - Thailand | Actual | Ordered Logit | +ve | NS | NS | NS | - | +ve | NS | - | - | NS | NS | NS | +ve | - | Health status, NS % older people (aged >65) in hh, NS  % with secondary education in hh, NS  Own land, NS House located near the coast, +ve House located near a river, NS  House located near a mountain, NS Area, +ve | |
| ^35^ | Multi-Hazards | Preparedness component: supply | Actual | Regression | - | - | - | - | - | - | - | - | - | - | - | - | - | >  See next column | Risk Rating / Risk Perception  Self-Family (e.g. Chemical Weapons), +ve  National (e.g. War), +ve  Natural (e.g. Earthquakes), NS  Accident (e.g. Road and Work), -ve  Industry (e.g. Industrial Accidents), NS  Technology (e.g. Power Outage), NS | |
|  |  | Preparedness component: skill | Actual | Regression | - | - | - | - | - | - | - | - | - | - | - | - | - | >  See next column | Risk Rating / Risk Perception  Self-Family (e.g. Chemical Weapons), NS  National (e.g. War), NS  Natural (e.g. Earthquakes), +ve Accident (e.g. Road and Work), +ve  Industry (e.g. Industrial Accidents), NS Technology (e.g. Power Outage), NS | |
|  |  | Preparedness component: planning | Actual | Regression | - | - | - | - | - | - | - | - | - | - | - | - | - | >  See next column | Risk Rating / Risk Perception  Self-Family (e.g. Chemical Weapons), NS  National (e.g. War), NS Natural (e.g. Earthquakes), NS  Accident (e.g. Road and Work), NS Industry (e.g. Industrial Accidents), NS  Technology (e.g. Power Outage), NS | |
|  |  | Preparedness component: protection. | Actual | Regression | - | - | - | - | - | - | - | - | - | - | - | - | - | >  See next column | Risk Rating / Risk Perception  Self-Family (e.g. Chemical Weapons), NS  National (e.g. War), NS Natural (e.g. Earthquakes), NS  Accident (e.g. Road and Work), NS Industry (e.g. Industrial Accidents), NS  Technology (e.g. Power Outage), NS | |
| ^36^ | Multi-Hazard Context | Preparedness: provisions/ supplies | Actual | Regression | NS | - | - | - | - | - | NS | - | - | NS | - | - | - | - | Frequency of Visits (Family Networks), +ve  Telephone Family /Relatives (Family Networks), NS Seek Family Advice (Family Networks), NS  Proximity to Relatives (Family Networks), +ve (A) State of Family Relations (Family Networks), NS | |
|  |  | Preparedness: skills | Actual | Regression | +ve | - | - | - | - | - | NS | - | - | NS | - | - | - | - | Frequency of Visits (Family Networks), +ve Telephone Family /Relatives (Family Networks), NS Seek Family Advice (Family Networks), NS  Proximity to Relatives (Family Networks), NS State of Family Relations (Family Networks), NS | |
|  |  | Preparedness: planning | Actual | Regression | NS | - | - | - | - | - | NS | - | - | NS | - | - | - | - | Frequency of Visits (Family Networks), NS Telephone Family /Relatives (Family Networks), NS  Seek Family Advice (Family Networks), NS  Proximity to Relatives (Family Networks), NS  State of Family Relations (Family Networks), -ve | |
|  |  | Preparedness: protection | Actual | Regression | +ve | - | - | - | - | - | NS | - | - | NS | - | - | - | - | Frequency of Visits (Family Networks), NS  Telephone Family /Relatives (Family Networks), NS  Seek Family Advice (Family Networks), NS Proximity to Relatives (Family Networks), -ve  State of Family Relations (Family Networks), NS | |
| ^37^ | Wildfire | Defence preparation | Actual | Regression | -ve | +ve | - | - | - | - | - | - | - | - | - | - | - | >  See next column | Risk Rating / Risk Perception  Risk likelihood, NS  Risk Severity, +ve  Other Factors  Area, +ve  Protection responsibility, NS  Warning reliability, NS  Lose water (no vs yes), -ve  Lose electricity (no vs yes), NS Lose mobile phone (no vs yes), NS  Lose water (NA vs yes), +ve  Lose electricity (NA vs yes) NS  Lose mobile phone (NA vs Yes) NS | |
|  |  | Evacuation preparation | Actual | Regression | -ve | +ve | - | - | - | - | - | - | - | - | - | - | - | >  See next column | Risk Rating / Risk Perception  Risk likelihood, NS  Risk Severity, +ve  Other Factors  Area, +ve Protection responsibility, +ve Warning reliability, NS Lose water (no vs yes), -ve  Lose electricity (no vs yes), -ve  Lose mobile phone (no vs yes), -ve  Lose water (NA vs yes), NS  Lose electricity (NA vs yes) NS  Lose mobile phone (NA vs Yes) NS | |
|  |  | House resilience | Actual | Regression | NS | +ve | - | - | - | - | - | - | - | - | - | - | - | >  See next column | Risk Rating / Risk Perception  Risk likelihood, NS  Risk Severity, +ve  Other Factors  Area, +ve  Protection responsibility, NS Warning reliability, -ve Lose water (no vs yes), -ve Lose electricity (no vs yes), -ve  Lose mobile phone (no vs yes), -ve  Lose water (NA vs yes), NS  Lose electricity (NA vs yes) NS  Lose mobile phone (NA vs Yes) NS | |
|  |  | Planning | Actual | Regression | -ve | +ve | - | - | - | - | - | - | - | - | - | - | - | >  See next column  > | Risk Rating / Risk Perception  Risk likelihood, NS  Risk Severity, +ve  Other Factors  Area, NS Protection responsibility, +ve  Warning reliability, NS  Lose water (no vs yes), -ve  Lose electricity (no vs yes), NS  Lose mobile phone (no vs yes), NS  Lose water (NA vs yes), NS  Lose electricity (NA vs yes) NS  Lose mobile phone (NA vs Yes) NS | |
| ^38^ | Earthquake & Tsunami | Disaster preparedness | Actual | Ordered logit | NS | -ve | - | - | - | +ve | -ve | NS | - | NS | NS | NS | NS | - | Head of household, NS Participated in evacuation drills and disaster education, NS Number of sources of information, +ve Education (one or more persons) +ve Number of members aged ≥ 60 years, -ve Number of members with a disability, +ve House location, NS Village characteristics:  Percentage of men with at least secondary education, NS Percentage of women with at least secondary education, +ve Percentage of members aged 65 years and over, NS Percentage of women, -ve | |
| ^39^ | Fire (Residential) | Presence of smoke detector | Actual | path analysis | - | - | - | - | - | - | - | - | - | - | - | - | +ve | >  See next column | Risk Rating / Risk Perception  Perceived Likelihood, NS | Other Factors  outcome expectancy, NS perceived knowledge, +ve self-efficacy, NS |
|  |  | Presence of fire extinguisher | Actual | path analysis | - | - | - | - | - | - | - | - | - | - | - | - | +ve | >  See next column | Risk Rating / Risk Perception  Perceived Likelihood, NS | Other Factors  outcome expectancy, +ve perceived knowledge, +ve self-efficacy, +ve |
|  |  | Presence of fire blanket | Actual | path analysis | - | - | - | - | - | - | - | - | - | - | - | - | NS | >  See next column | Risk Rating / Risk Perception  Perceived Likelihood, +ve | Other Factors  outcome expectancy, NS  perceived knowledge, +ve self-efficacy, NS |
| ^40^ | Earthquakes & Household Fires | Overall preparedness, earthquake preparedness, & fire preparedness) | Actual | Multivariate & univariate | - | - | - | - | - | - | - | - | - | - | - | - | - | - | (Note, Table 8 Significant predictors **only** of preparedness - multivariate analysis) Intervention (A), Control (B)  Dependent Variable: Predictors overall preparedness Self-efficacy, +ve (A & B) Collective efficacy, -ve (A & B) Anxiety (earthquake), +ve (A & B) Trust, +ve (A), -ve (B)  Dependent Variable: Predictors of earthquake preparedness Anxiety (earthquake), , +ve (A & B) Trust, -ve (A & B)  Dependent Variable: Predictors of fire preparedness Self-efficacy, -ve (A), +ve (B) | |
| ^41^ | Multi-Hazard Context | Preparedness | Actual | Regression | - | - | - | - | - | - | - | - | - | - | - | - | - | - | Regressions for Supply (A), Skills (B), Plans (C), Protect (D)  Family Based Networks, +ve (A), NS (B), NS (C), +ve (D)  Micro-Neighborhood Network, +ve (A), NS (B), +ve (C), NS (D) Macro-Community Networks, +ve (A), +ve (B), NS (C), NS (D) | |
| ^42^ | "Natural disaster" | Collective protection | Actual | Chi-square test of independence | - | - | - | - | - | - | - | - | - | - | - | - | - | - | There are statistically significant differences in responses between public administration employees and respondents who are not public administration employees (residents) to the question: - concerning the shelters in the residence (p = 0.059); - concerning the evacuation rules (p = 0.000); - concerning the hygiene and health care (p = 0.002); - concerning the appropriate toiletries (p = 0.029); - concerning the behaviour in the evacuation shelter (p = 0.042) | |
| ^43^ | Earthquake | Actual adoption of hazard adjustment activities & the likelihood of adopting these hazard adjustment activities | Actual | Regression | - | - | - | - | - | - | - | - | - | - | - | - | - | - | (Note, see Table 5 for full list of DV's e.g. use of Latches) DV: Strap Item (A), Plan (B), Insurance (C) - adoption intentions  Protect persons effectively, +ve (A & B), NS (C) Protect property effectively, NS (A & B), +ve (C) Cost Money, NS (A, B & C) Require knowledge, NS (A, B & C) Require effort, -ve (A), NS (B & C) Require cooperation, +ve (A), NS (A & B) Useful for other hazards, +ve (A, B & C) | |
| ^44^ | "Natural disaster" | Preparedness behaviours | Actual | Multivariate Regression | - | - | - | - | - | - | - | - | - | - | - | - | - | - | Self-preparedness responsibility, +ve | |
| ^45^ | Terrorism | Preparedness | Actual | Poisson Regression | - | - | - | - | - | - | - | - | - | - | - | - | - | >  See next column | Risk Rating / Risk Perception  Severity, NS  Vulnerability, +ve  Anxiety, -ve  Other Factors  Self-efficacy, +ve | |
| ^46^ | "Natural disaster" (e.g. earthquakes and tsunamis) | Household preparedness | Actual | Logit | +ve | +ve | - | NS | NS | +ve | NS | - | - | - | NS | NS | - | - | VDPO, NS Living with someone aged 75 or older, NS Living with those requiring special assistance, NS Concerned about a natural disaster, NS | |
| ^47^ | Disasters | Preparedness (deemed to be ‘‘prepared’’) | Actual | Logit | - | +ve | - | - | NS | NS | NS | +ve | NS | - | - | -ve | - | - | Medical factors (List), +ve & NS Unable to afford a doctor in the past year, NS  Subjective Preparedness (List), +ve | |
| ^48^ | Disasters (notes earthquakes, typhoons, infectious diseases) | Household preparedness | Actual | Logit | NS | NS | - | - | NS | -ve | - | +ve | - | - | - | - | +ve | NS | Risk Rating / Risk Perception  Note: Risk awareness score  Other Factors  Regions, +ve Participation in emergency training - last year, +ve Emergency knowledge score, +ve Attitudes towards emergency preparedness, +ve Self-reliance, +ve Fate submissiveness, +ve | |
| ^49^ | Disasters (Flooding, Hail, High Winds, and Hurricanes) | Preparedness | Actual | OLS | NS | +ve | - | - | - | - | - | +ve | NS | - | - | - | +ve | - |  | |
| ^50^ | Natural Hazards | Preparedness & Intention | Actual | Hierarchical Multiple Regression | +ve | NS | - | - | - | - | - | - | - | - | - | NS | - | - | Aust/NZ (Location), +ve Participation (Community), NS Children hazard education, NS Confidence (Government), NS Collective efficacy, NS  Empowerment, NS Trust, NS Distrust, NS Negative outcome expectancy, -ve Positive outcome expectancy, NS  Responsibility for others, NS  Personal responsibility, +ve | |
| ^51^ | Flooding | Preparations | Actual | Regression | - | - | - | - | - | - | - | - | - | - | - | - | - | >  See next column | Risk Rating / Risk Perception  Probability, +ve  Consequences, +ve | Other Factors  Affect, -ve  Response efficacy, NS Participation, +ve  Community efficacy, NS Trust, NS  Empowerment, NS |
| ^52^ | Pandemic Influenza | Recommended behaviours (affective response) | Actual | Structural Equation Modelling | - | - | - | - | - | - | - | - | - | - | - | - | - | >  See next column | Risk Rating / Risk Perception  Likelihood, +ve  Severity, +ve  Affective Response (worry about pandemic), +ve  Affective Response (worry about pandemic), +ve  Other Factors  Level of preparedness of institutions, NS | |
| ^53^ | "Natural disaster" | Disaster survival kit (decision stages, unaware to have a kit) | Actual | Ordered Logit | - | - | - | - | - | - | - | - | - | - | - | - | - | >  See next column | Note: Predictors of changes in stages for providing disaster survival kit  Risk Rating / Risk Perception  Perceived susceptibility, NS Perceived severity, NS  Other Factors Perceived benefits, +ve  Perceived barriers, -ve Self-efficacy, NS | |
| ^54^ | Volcanic | Household emergency preparedness | Actual | OLS | NS | NS | NS | NS | - | NS | - | NS | NS | - | - | - | - | NS | Hazard intrusiveness, NS  Affective response, NS Community boundedness, +ve  Past information search, +ve Lahar zone location, NS Crater proximity, +ve | |

Note: As a result of the format of Kyne et al. [1], see Kyne et al. figure five, we are unable to report the findings according to our table format. Please see Kyne et al. [1] original paper for the full set of analysis. Kyne et al [1]: Hurricane - Subjective & objective preparedness.

REFERENCES

1. Kirschenbaum A (Avi), Rapaport C, Canetti D. The impact of information sources on earthquake preparedness. *Int J Disaster Risk Reduct*. 2017;21(July 2016):99-109. doi:10.1016/j.ijdrr.2016.10.018

2. Basolo V, Steinberg LJ, Burby RJ, Levine J, Cruz AM, Huang C. The effects of confidence in government and information on perceived and actual preparedness for disasters. *Environ Behav*. 2009;41(3):338-364. doi:10.1177/0013916508317222

3. Basolo V, Steinberg LJ, Gant S. Hurricane threat in Florida: examining household perceptions, beliefs, and actions. *Environ Hazards*. 2017;16(3):253-275. doi:10.1080/17477891.2016.1277968

4. Corwin KA, Brand BD, Hubbard ML, Johnston DM. Household preparedness motivation in lahar hazard zones: assessing the adoption of preparedness behaviors among laypeople and response professionals in communities downstream from Mount Baker and Glacier Peak (USA) volcanoes. *J Appl Volcanol*. 2017;6(1):1-19. doi:10.1186/s13617-017-0055-8

5. Landahl M, Cox C. Beyond the Plan: Individual Responder and Family Preparedness in the Resilient Organization. *Homel Secur Aff*. 2009;5(3):1-22.

6. Bourque LB, Regan R, Kelley MM, Wood MM, Kano M, Mileti DS. An Examination of the Effect of Perceived Risk on Preparedness Behavior. *Environ Behav*. 2013;45(5):615-649. doi:10.1177/0013916512437596

7. Henly-Shepard S, Anderson C, Burnett K, Cox LJ, Kittinger JN, Ka‘aumoana M. Quantifying household social resilience: a place-based approach in a rapidly transforming community. *Nat Hazards*. 2015;75(1):343-363. doi:10.1007/s11069-014-1328-8

8. Cope MR, Lee MR, Slack T, et al. Geographically distant social networks elevate perceived preparedness for coastal environmental threats. *Popul Environ*. 2018;39(3):277-296. doi:10.1007/s11111-017-0292-0

9. Adame B, Miller CH. Vested interest: developing scales for assessing flooding preparedness. *Disaster Prev Manag An Int J*. 2016;25(3):282-297. doi:10.1108/DPM-08-2015-0196

10. Bradford RA, O’Sullivan JJ, Van Der Craats IM, et al. Risk perception - Issues for flood management in Europe. *Nat Hazards Earth Syst Sci*. 2012;12(7):2299-2309. doi:10.5194/nhess-12-2299-2012

11. DeBastiani SD, Strine TW, Vagi SJ, Barnett DJ, Kahn EB. Preparedness Perceptions, Sociodemographic Characteristics, and Level of Household Preparedness for Public Health Emergencies: Behavioral Risk Factor Surveillance System, 2006-2010. *Heal Secur*. 2015;13(5):317-326. doi:10.1089/hs.2014.0093

12. Donahue AK, Eckel CC, Wilson RK. Ready or Not? How Citizens and Public Officials Perceive Risk and Preparedness. *Am Rev Public Adm*. 2014;44(4S):89S –111S. doi:10.1177/0275074013506517

13. Ranjbar M, Soleimani AA, Sedghpour BS, Shahboulaghi FM, Paton D, Noroozi M. The predictors of earthquake preparedness in Tehran households Maryam. *Electron Physician*. 2018;10(3):6478-6486. doi:http://dx.doi.org/10.19082/6478

14. Oriangi G, Albrecht F, Di Baldassarre G, et al. Household resilience to climate change hazards in Uganda. *Int J Clim Chang Strateg Manag*. 2019;12(1):59-73. doi:10.1108/IJCCSM-10-2018-0069

15. Kruger J, Chen B, Heitfeld S, Witbart L, Bruce C, Pitts DL. Attitudes, Motivators, and Barriers to Emergency Preparedness Using the 2016 Styles Survey. *Health Promot Pract*. 2018;Vol. XX(No. (X)):1-9. doi:10.1177/1524839918794940

16. Han Z, Lu X, Hörhager EI, Yan J. The effects of trust in government on earthquake survivors’ risk perception and preparedness in China. *Nat Hazards*. 2017;86(1):437-452. doi:10.1007/s11069-016-2699-9

17. Annis H, Jacoby I, De Mers G. Disaster preparedness among active duty personnel, retirees, veterans, and dependents. *Prehosp Disaster Med*. 2016;31(2):132-140. doi:10.1017/S1049023X16000157

18. Knuth D, Schulz S, Kietzmann D, Stumpf K, Schmidt S. Better safe than sorry - Emergency knowledge and preparedness in the German population. *Fire Saf J*. 2017;93(September):98-101. doi:10.1016/j.firesaf.2017.08.003

19. Hung LS. Comparing spousal agreement on perceived responsibility for household natural hazard preparedness to actual behavior. *PLoS One*. 2019;14(8):1-19. doi:10.1371/journal.pone.0221217

20. Mabuku MP, Senzanje A, Mudhara M, Jewitt G, Mulwafu W. Rural households’ flood preparedness and social determinants in Mwandi district of Zambia and Eastern Zambezi Region of Namibia. *Int J Disaster Risk Reduct*. 2018;28(June 2018):284-297. doi:10.1016/j.ijdrr.2018.03.014

21. Oral M, Yenel A, Oral E, Aydin N, Aydin N. Earthquake experience and preparedness in Turkey. *Disaster Prev Manag An Int J*. 2015;24(1):21-37. doi:10.1108/DPM-01-2013-0008

22. Nguyen LH, Shen H, Ershoff D, Afifi AA, Bourque LB. Exploring the causal relationship between exposure to the 1994 Northridge earthquake and pre- and post- earthquake preparedness activities. *Earthq Spectra*. 2006;22(3):569-587. doi:10.1193/1.2219108

23. Bodas M, Siman-Tov M, Kreitler S, Peleg K. The Role of Victimization in Shaping Households’ Preparedness for Armed Conflicts in Israel. *Disaster Med Public Health Prep*. 2018;12(1):67-75. doi:10.1017/dmp.2017.38

24. Ejeta LT, Ardalan A, Paton D, Yaseri M. Predictors of community preparedness for flood in Dire-Dawa town, Eastern Ethiopia: Applying adapted version of Health Belief Model. *Int J Disaster Risk Reduct*. 2016;19(October 2016):341-354. doi:10.1016/j.ijdrr.2016.09.005

25. Espina E, Teng-calleja M. A Social Cognitive Approach to Disaster Preparedness. *Philipp J Psycology*. 2015;48(2):161-174. https://www.pap.org.ph/includes/view/default/uploads/pjp-journals/PJP1502_Final_7Espina_and_Calleja.pdf

26. DeYoung SE, Lewis DC, Seponski DM, Augustine DA, Phal M. Disaster preparedness and well-being among Cambodian– and Laotian–Americans. *Disaster Prev Manag An Int J*. 2019;(NO.ahead-of-print). doi:10.1108/DPM-01-2019-0034

27. Wei HH, Sim T, Han Z. Confidence in authorities, neighborhood cohesion and natural hazards preparedness in Taiwan. *Int J Disaster Risk Reduct*. 2019;40(November 2019):101265. doi:10.1016/j.ijdrr.2019.101265

28. Wood MM, Mileti DS, Kano M, Kelley MM, Regan R, Bourque LB. Communicating Actionable Risk for Terrorism and Other Hazards. *Risk Anal*. 2012;32(4):601-615. doi:10.1111/j.1539-6924.2011.01645.x

29. Bodas M, Siman-Tov M, Kreitler S, Peleg K. Assessment of Emergency Preparedness of Households in Israel for War - Current Status. *Disaster Med Public Health Prep*. 2015;9(4):382-390. doi:10.1017/dmp.2015.56

30. DeYoung S, Peters M. My community, my preparedness: The role of sense of place, community, and confidence in government in disaster readiness. *Int J Mass Emerg Disasters*. 2016;34(2):250-282.

31. Baytiyeh H, Naja M. The effects of fatalism and denial on earthquake preparedness levels. *Disaster Prev Manag An Int J*. 2016;25(2):154-167. doi:10.1108/DPM-07-2015-0168

32. Rebmann T, Strawn AM, Swick Z, Reddick D. Personal Disaster and Pandemic Preparedness of U.S. Human Resource Professionals. *J Biosaf Heal Educ*. 2013;1(1):1-7. doi:10.4172/2332-0893.1000102

33. Xu D, Peng L, Liu S, Wang X. Influences of Risk Perception and Sense of Place on Landslide Disaster Preparedness in Southwestern China. *Int J Disaster Risk Sci*. 2018;9(2):167-180. doi:10.1007/s13753-018-0170-0

34. Hoffmann R, Muttarak R. Learn from the Past, Prepare for the Future: Impacts of Education and Experience on Disaster Preparedness in the Philippines and Thailand. *World Dev*. 2017;69(August 2017):32-51. doi:10.1016/j.worlddev.2017.02.016

35. Kirschenbaum A. Preparing For The Inevitable: Environmental Risk Perceptions and Disaster Preparedness. *Int J Mass Emerg Disasters*. 2005;23(2):97-127.

36. Kirschenbaum A. Families and Disaster Behavior: A Reassessment of Family Preparedness Alan. *Int J Mass Emerg Disasters*. 2006;24(1):111-143.

37. McNeill IM, Dunlop PD, Heath JB, Skinner TC, Morrison DL. Expecting the unexpected: Predicting physiological and psychological wildfire preparedness from perceived risk, responsibility, and obstacles. *Risk Anal*. 2013;33(10):1829-1843. doi:10.1111/risa.12037

38. Muttarak R, Pothisiri W. The role of education on disaster preparedness: Case study of 2012 Indian Ocean earthquakes on Thailand’s Andaman coast. *Ecol Soc*. 2013;18(4):1-16. doi:10.5751/ES-06101-180451

39. Stumpf K, Knuth D, Kietzmann D, Schmidt S. Adoption of fire prevention measures – Predictors in a representative German sample. *Saf Sci*. 2017;94(April 2017):94-102. doi:10.1016/j.ssci.2016.12.023

40. Joffe H, Perez-Fuentes G, Potts HWW, Rossetto T. How to increase earthquake and home fire preparedness: the fix-it intervention. *Nat Hazards*. 2016;84(3):1943-1965. doi:10.1007/s11069-016-2528-1

41. Kirschenbaum A. Generic sources of disaster communities: A social network approach. *Int J Sociol Soc Policy*. 2004;24(10-11):94-129. doi:10.1108/01443330410791073

42. Tej J, Živčák P, Ali Taha V, Sirkova M. Crisis Awareness of the Municipal District Residents: Implication for Crisis Management At the Local Government Level. *Qual Innov Prosper*. 2014;18(2):1-14. doi:10.12776/qip.v18i2.295

43. Wu HC, Greer A, Murphy HC, Chang R. Preparing for the new normal: Students and earthquake hazard adjustments in Oklahoma. *Int J Disaster Risk Reduct*. 2017;25(October 2017):312-323. doi:10.1016/j.ijdrr.2017.09.033

44. Yong AG, Lemyre L. Getting Canadians prepared for natural disasters: a multi-method analysis of risk perception, behaviors, and the social environment. *Nat Hazards*. 2019;98(1):319-341. doi:10.1007/s11069-019-03669-2

45. Wirtz PW, Rohrbeck CA, Burns KM. Anxiety effects on disaster precautionary behaviors: A multi-path cognitive model. *J Health Psychol*. 2019;24(10):1401-1411. doi:10.1177/1359105317720277

46. Tomio J, Sato H, Matsuda Y, Koga T, Mizumura H. Household and Community Disaster Preparedness in Japanese Provincial City: A Population-Based Household Survey. *Adv Anthropol*. 2014;4(2):68-77. doi:10.4236/aa.2014.42010

47. Ablah E, Konda K, Kelley CL. Factors predicting individual emergency preparedness: A multi-state analysis of 2006 BRFSS data. *Biosecurity and Bioterrorism*. 2009;7(3):317-330. doi:10.1089/bsp.2009.0022

48. Chen CY, Xu W, Dai Y, et al. Household preparedness for emergency events: A cross-sectional survey on residents in four regions of China. *BMJ Open*. 2019;9(11):1-9. doi:10.1136/bmjopen-2019-032462

49. Donner WR, Lavariega-Montforti J. Ethnicity, income, and disaster preparedness in Deep South Texas, United States. *Disasters*. 2018;42(4):719-733. doi:10.1111/disa.12277

50. Kelly B, Ronan KR. Preparedness for natural hazards: Testing an expanded education- and engagement-enhanced social cognitive model. *Nat Hazards*. 2018;91(1):19-35. doi:10.1007/s11069-017-3093-y

51. Kerstholt J, Duijnhoven H, Paton D. Flooding in The Netherlands: How people’s interpretation of personal, social and institutional resources influence flooding preparedness. *Int J Disaster Risk Reduct*. 2017;24(September 2017):52-57. doi:10.1016/j.ijdrr.2017.05.013

52. Prati G, Pietrantoni L, Zani B. A Social-Cognitive Model of Pandemic Influenza H1N1 Risk Perception and Recommended Behaviors in Italy. *Risk Anal*. 2011;31(4):645-656. doi:10.1111/j.1539-6924.2010.01529.x

53. Jassempour K, Shirazi KK, Fararooei M, Shams M, Shirazi AR. The impact of educational intervention for providing disaster survival kit: Applying precaution adoption process model. *Int J Disaster Risk Reduct*. 2014;10(December 2014):374-380. doi:10.1016/j.ijdrr.2014.10.012

54. Wei HL, Lindell MK. Washington households’ expected responses to lahar threat from Mt. Rainier. *Int J Disaster Risk Reduct*. 2017;22(June 2017):77-94. doi:10.1016/j.ijdrr.2016.10.014
