# Supplementary material for: An assessment of nurses’ perceived and actual household emergency preparedness
Source: PLoS One. 2024 Apr 18;19(4):e0300536. doi: 10.1371/journal.pone.0300536 (PMC11025835; doi:10.1371/journal.pone.0300536)
Supplement: S2 Table — (DOCX) [file pone.0300536.s003.docx]

**An assessment of nurses’ perceived and actual household emergency preparedness**

Gavin David Brown^1 *^

Caroline McMullan ^1^

Ann Largey ^1^

David Leon ^1^

* Corresponding Author

E-mail address: [gavin.brown@dcu.ie](mailto:gavin.brown@dcu.ie)

**S2 Table Summary of the studies that examined perceived and actual preparedness using regression analysis**

Notes: +ve = Positive and significant relationship, -ve = negative and significant relationship, NS = non-significant.

| **Ref** | **Disaster** | **Preparedness Variable** | **Model** | **Analysis** | **Female** | **Age** | **Owns Home** | **Years of residence** | **Urban (Inc. Town)** | **Education** | **Children** | **Income** | **Race** | **Marital Status** | **Household Size** | **Employed** | **Disaster Exposure** | **Risk Rating / Risk Perception** | **Other Factors** |
| --- | --- | --- | --- | --- | --- | --- | --- | --- | --- | --- | --- | --- | --- | --- | --- | --- | --- | --- | --- |
| ^1^ | Earthquake | Actual preparedness | Actual | Regression | NS | NS | - | - | - | +ve | - | - | - | - | - | - | +ve | - | Living in risk zone, NS  Religion, -ve  Trust in information source, NS  Past experience*trust, NS |
|  |  | Actual preparedness for Israeli Jews | Actual | Regression | NS | NS | - | - | - | NS | - | - | - | - | - | - | +ve | - | Living in risk zone, NS  Trust in information source, NS  Past experience*trust, -ev |
|  |  | Actual preparedness for Israeli Arabs | Actual | Regression | NS | NS | - | - | - | +ve | - | - | - | - | - | - | NS | - | Living in risk zone, NS  Trust in information source, NS  Past experience*trust, +ev |
|  |  | Perceived preparedness | Perceived | Regression | +ve | -ve | - | - | - | NS | - | - | - | - | - | - | NS | - | Living in a risk zone, +ve Religion, NS Trust in information source, NS  Past experience*trust, NS |
|  |  | Perceived preparedness for Israeli Jews | Perceived | Regression | +ve | -ve | - | - | - | NS | - | - | - | - | - | - | NS | - | Living in risk zone, NS  Trust in information source, NS  Past experience*trust, NS |
|  |  | Perceived preparedness for Israeli Arabs | Perceived | Regression | NS | -ve | - | - | - | NS | - | - | - | - | - | - | NS | - | Living in risk zone, +ev  Trust in information source, NS  Past experience*trust, NS |
| ^2^ | Earthquakes / Hurricane | Perceived level of preparedness for earthquakes | Perceived | Logit | NS | NS | NS | - | - | NS | NS | NS | NS | NS | - | - | NS | >  See next column | Risk Rating / Risk Perception  Dread, NS Fatal, NS Happen, NS  Other Factors  Confidence in government, +ve Information sources, +ve |
|  |  | Perceived level of preparedness for hurricanes | Perceived | Logit | NS | NS | NS | - | - | NS | NS | NS | NS | NS | - | - | NS | >  See next column | Risk Rating / Risk Perception  Dread, NS Fatal, -ve Happen, NS  Other Factors  Confidence in government, +ve Information sources, +ve |
|  | Earthquakes | Preparedness actions for earthquakes - family plan | Actual | Logit | NS | NS | NS | - | - | NS | NS | NS | NS | NS | - | - | NS | >  See next column | Risk Rating / Risk Perception  Dread, NS Fatal, NS Happen, NS  Other Factors  Confidence in government, NS Information sources, +ve |
|  |  | Preparedness actions for earthquakes - supplies | Actual | Logit | NS | NS | NS | - | - | NS | NS | NS | NS | NS | - | - | NS | >  See next column | Risk Rating / Risk Perception  Dread, NS Fatal, NS Happen, NS  Other Factors  Confidence in government, NS Information sources, NS |
|  |  | Preparedness actions for earthquakes - mitigation | Actual | Logit | NS | NS | NS | - | - | NS | NS | NS | NS | NS | - | - | NS | >  See next column | Risk Rating / Risk Perception  Dread, NS Fatal, NS Happen, NS  Other Factors  Confidence in government, NS Information sources, NS |
|  |  | Preparedness actions for earthquakes - shut off utilities | Actual | Logit | NS | NS | +ve | - | - | NS | NS | NS | NS | NS | - | - | NS | >  See next column | Risk Rating / Risk Perception  Dread, NS Fatal, NS Happen, NS  Other Factors  Confidence in government, NS Information sources, +ve |
|  | Hurricane | Preparedness actions for hurricanes - family plan | Actual | Logit | NS | NS | NS | - | - | NS | NS | NS | NS | +ve | - | - | NS | >  See next column | Risk Rating / Risk Perception  Dread, +ve Fatal, NS Happen, NS  Other Factors  Confidence in government, +ve Information sources, NS |
|  |  | Preparedness actions for hurricanes - supplies | Actual | Logit | NS | NS | +ve | - | - | NS | +ve | NS | NS | NS | - | - | NS | >  See next column | Risk Rating / Risk Perception  Dread, NS Fatal, NS Happen, NS  Other Factors  Confidence in government, NS Information sources, NS |
|  |  | Preparedness actions for hurricanes - shut off utilities | Actual | Logits | -ve | +ve | NS | - | - | NS | NS | NS | NS | NS | - | - | NS | >  See next column | Risk Rating / Risk Perception  Dread, NS Fatal, NS Happen, NS  Other Factors  Confidence in government, NS Information sources, NS |
| ^3^ | Hurricane | Perceived level of preparedness | Perceived | Ordered Logit | NS | +ve | NS | - | - | NS | NS | NS | NS | NS | - | - | NS | +ve | Immigrant, -ve Information, NS Perceived readiness of local government, +ve |
|  |  | House preparedness (sandbags on site) | Actual | Logit | -ve | NS | NS | - | - | NS | +ve | NS | -ve | +ve | - | - | NS | +ve | Immigrant, NS Information, NS Perceived readiness of local government, NS |
|  |  | Household preparedness (family plan) | Actual | Logit | NS | NS | NS | - | - | NS | NS | NS | NS | NS | - | - | +ve | NS | Information (a lot), +ve Perceived readiness of local government, +ve |
|  |  | Preparedness (all recommended items on hand) | Actual | Logit | NS | NS | +ve | - | - | NS | NS | NS | NS | +ve | - | - | NS | NS | Immigrant, NS Information (a lot), NS Perceived readiness of local government, NS |
| ^7^ | Natural Hazard - Multi-Hazard Context | Household perceived preparedness | Perceived | Regression | NS | - | NS | NS | - | - | - | - | - | - | - | - | - | - | Know how to fish, NS Know how to preserve food, NS Origin: Mainland, NS Trade fish, NS The profession - Teacher, +ve Have a safe place to evacuate to, NS Know of safe places to evacuate to, NS Know of public awareness programs, NS Think early warning systems are very effective, NS |
|  |  |  |  |  | NS | NS | - | -ve | - | - | - | - | - | - | - | - | NS | - | The profession - Teacher, NS Have a safe place to evacuate to, +ve Participation in a Community Group, NS |
|  |  |  |  |  | NS | NS | - | -ve | - | - | - | NS | - | - | - | - | +ve | - | Have a safe place to evacuate to, NS |
|  |  |  |  |  | NS | NS | - | -ve | - | - | - | - | NS | - | - | - | - | - | Know of public awareness programs, +ve Think early warning systems are somewhat  effective, NS |
|  |  | Coping capacity (e.g. stocked food/water supplies/emergency rations) | Actual | Regression | NS | - | - | -ve | - | NS | - | NS | - | - | - | - | - | - | Perceived as Prepared, NS Feel the community is prepared, NS Origin, NS Participation in a Community Group, +ve |
|  |  |  |  |  | NS | - | - | NS | - | - | - | - | - | - | - | - | - | - | Perceived as Prepared, NS Feel the community is prepared, +ve Origin, NS |
| ^11^ | Public Health Emergencies | Level of perceived preparedness | Perceived | Logit | -ve | +ve | - | - | - | -ve | - | - | +ve | - | - | - | - | - | Survey language, English, +ve |
|  |  | Number of preparedness items | Actual | Logit | -ve | +ve | - | - | - | -ve | - | - | -ve | - | - | - | - | - | (5-Items)  Survey language, English, +ve |
| ^13^ | Earthquake | Perceived preparedness | Perceived | Multivariate Regression | - | - | - | - | - | - | - | - | - | - | - | NS | NS | - | Trust, +ve  Residence region, NS Family members, -ve |
|  |  | Actual preparedness behaviour | Actual | Multivariate Regression | -ve | NS | NS | - | - | - | - | - | - | - | - | - | NS | - | Trust, +ve  Family members, +ve Monthly income, +ve |
