# Supplementary material for: An assessment of nurses’ perceived and actual household emergency preparedness
Source: PLoS One. 2024 Apr 18;19(4):e0300536. doi: 10.1371/journal.pone.0300536 (PMC11025835; doi:10.1371/journal.pone.0300536)
Supplement: S3 Table — (DOCX) [file pone.0300536.s004.docx]

**An assessment of nurses’ perceived and actual household emergency preparedness**

Gavin David Brown^1 *^

Caroline McMullan ^1^

Ann Largey ^1^

David Leon ^1^

* Corresponding Author

E-mail address: [gavin.brown@dcu.ie](mailto:gavin.brown@dcu.ie)

S3.1 Table Regression Analysis of Perceived and Actual Preparedness

|  | **Dependent Variable: Household Preparedness Score (Z-Score) †** | | |  | **Dependent Variable: Perceived Preparedness ‡** | | |
| --- | --- | --- | --- | --- | --- | --- | --- |
| **Explanatory variables** | **Coef.** | **Robust Std. Err.** | **P** |  | **Coef.** | **Robust Std. Err.** | **P** |
| Female | 0.032 | 0.149 | 0.829 |  | 0.305 | 0.263 | 0.245 |
| Age 35-44 | 0.039 | 0.117 | 0.740 |  | -0.313  0.162 | 0.249 | 0.208 |
| Age 45 & Older | -0.073 | 0.161 | 0.651 |  | -0.409 | 0.270 | 0.129 |
| Live – village | -0.236 | 0.139 | 0.091 |  | -0.315 | 0.261 | 0.228 |
| Live – town | -0.236 | 0.110 | 0.563 |  | -0.467* | 0.215 | 0.039 |
|  |  |  |  |  | 0.389ǂ |  | 0.070 |
| Live – suburbs/outskirts of a city | -0.412*** | 0.125 | 0.001 |  | -0.145 | 0.223 | 0.515 |
| Live – city | -0.410** | 0.147 | 0.006 |  | -0.657** | 0.246 | 0.007 |
| Owns home | 0.511*** | 0.109 | 0.001 |  | 0.400** | 0.173 | 0.021 |
| Years at address | 0.003 | 0.006 | 0.626 |  | 0.010 | 0.010 | 0.296 |
| Number of adults at household | 0.061 | 0.046 | 0.191 |  | 0.019 | 0.085 | 0.827 |
| Number of children (age <18) at household | -0.035 | 0.037 | 0.346 |  | -0.189** | 0.066 | 0.004 |
| Income 30,000-70,000 | 0.483** | 0.160 | 0.003 |  | 0.449 | 0.291 | 0.123 |
| Income Over 70,000 | 0.707*** | 0.177 | 0.001 |  | 0.424 | 0.322 | 0.188 |
| Years practicing | 0.003 | 0.006 | 0.613 |  | -0.004 | 0.011 | 0.695 |
| Risk rating Max: socio-natural | -0.013 | 0.009 | 0.173 |  | -0.021 | 0.017 | 0.217 |
| Risk rating Max: technological | 0.008 | 0.012 | 0.520 |  | 0.003 | 0.021 | 0.873 |
| Risk rating Max: civil | 0.003 | 0.011 | 0.754 |  | -0.020 | 0.019 | 0.293 |
| Exposure to socio-natural emergencies | 0.075* | 0.033 | 0.025 |  | 0.166** | 0.061 | 0.007 |
| Exposure to technological emergencies | 0.072 | 0.064 | 0.266 |  | 0.142 | 0.102 | 0.164 |
| Exposure to civil emergencies | 0.019 | 0.035 | 0.597 |  | -0.009 | 0.063 | 0.883 |
| Constant | -1.130*** | -3.66 | 0.001 |  |  | | |
| Cut 1 |  | | |  | -0.457 | 0.559 |  |
| Cut 2 |  | | |  | 1.664 | 0.564 |  |
| R^2^ | .264 | | |  |  | | |
| F | 5.39*** | | |  |  | | |
| Wald Chi-squared (df = 20) |  | | |  | χ2=50.20*** | | |
| N | 322 | | |  | 298 | | |

Notes: † OLS Regression; ‡ Ordered Probit Regression; $ǂ p\leq0.1, *p<0.05, **p<0.01, ***p<0.001$

S3.2 Table Marginal Effects

|  | **Dependent Variable: Perceived Preparedness** | | | | | |
| --- | --- | --- | --- | --- | --- | --- |
|  | **Marginal Effects (dy/dx)** | | | | | |
|  | **No** | **P** | **Somewhat** | **P** | **Yes** | **P** |
| Female | -0.065 | 0.249 | -0.004 | 0.680 | 0.070 | 0.246 |
| Age 35-44 | 0.067 | 0.211 | -0.104* | 0.040 | 0.037 | 0.511 |
| Age 45 & Older | 0.087 | 0.135 | 0.006 | 0.671 | -0.093 | 0.130 |
| Live – village | 0.067 | 0.232 | 0.005 | 0.676 | -0.072 | 0.226 |
| Live – town | 0.100* | 0.038 | -0.189*** | 0.001 | 0.089ǂ | 0.071 |
| Live – suburbs/outskirts of a city | 0.031 | 0.516 | 0.002 | 0.709 | -0.033 | 0.513 |
| Live – city | 0.140** | 0.010 | 0.010 | 0.665 | -0.150** | 0.009 |
| Owns home | -0.085* | 0.021 | -0.006 | 0.674 | 0.091* | 0.025 |
| Years at address | -0.002 | 0.301 | 0.000 | 0.678 | 0.002 | 0.294 |
| Number of adults at household | -0.004 | 0.827 | 0.000 | 0.843 | 0.004 | 0.827 |
| Number of children (age <18) at household | 0.040** | 0.004 | 0.003 | 0.669 | -0.043** | 0.005 |
| Income 30,000-70,000 | -0.096 | 0.129 | -0.007 | 0.666 | 0.103 | 0.120 |
| Income Over 70,000 | -0.091 | 0.193 | -0.006 | 0.670 | 0.097 | 0.184 |
| Years practicing | 0.001 | 0.695 | 0.000 | 0.767 | -0.001 | 0.695 |
| Risk rating max: socio-natural | 0.004 | 0.219 | 0.000 | 0.677 | -0.005 | 0.215 |
| Risk rating max: tech. | -0.001 | 0.873 | 0.000 | 0.882 | 0.001 | 0.873 |
| Risk rating: civil | 0.004 | 0.292 | 0.000 | 0.694 | -0.005 | 0.297 |
| Exposure to socio-natural emergencies | -0.035** | 0.009 | -0.002 | 0.661 | 0.038** | 0.006 |
| Exposure to tech. emergencies | -0.030 | 0.169 | -0.002 | 0.676 | 0.032 | 0.167 |
| Exposure to civil emergencies | 0.002 | 0.884 | 0.000 | 0.884 | -0.002 | 0.883 |

Notes: Marginal effects are estimated using the mean values for all other explanatory variables

$$ǂ p\leq0.1, *p<0.05, **p<0.01, ***p<0.001$$
